# Pharmacokinetics, Safety and Cognitive Function Profile of Rupatadine 10, 20 and 40 mg in Healthy Japanese Subjects: A Randomised Placebo-Controlled Trial

**DOI:** 10.1371/journal.pone.0163020

**Published:** 2016-09-15

**Authors:** Jörg Täubel, Georg Ferber, Sara Fernandes, Ulrike Lorch, Eva Santamaría, Iñaki Izquierdo

**Affiliations:** 1 Richmond Pharmacology Ltd, St George's University of London, Cranmer Terrace, London, United Kingdom; 2 Statistik Georg Ferber GmbH, Cagliostrostrasse, Riehen, Switzerland; 3 J. Uriach y Compañía, S.A., Avda. Camí Reial, Barcelona, Spain; Tongji Hospital of Tongji Medical College of Huazhong University of Science and Technology, CHINA

## Abstract

**Introduction:**

Rupatadine is a marketed second generation antihistamine, with anti-PAF activity, indicated for symptomatic treatment of allergic rhinitis and urticaria. This study was conducted to evaluate the pharmacokinetics (PK), pharmacodynamics (PD), safety and tolerability of rupatadine in healthy Japanese subjects after single and multiple oral doses.

**Methods:**

In this randomised, double-blind, placebo-controlled study, 27 male and female healthy Japanese subjects were administered single and multiple escalating rupatadine dose of 10, 20 and 40 mg or placebo. Blood samples were collected at different time points for PK measurements and subjects were assessed for safety and tolerability. The effect of rupatadine on cognitive functioning was evaluated by means of computerized cognitive tests: rapid visual information processing (RVP), reaction time (RT), spatial working memory (SWM) and visual analogue scales (VAS).

**Results:**

Exposure to rupatadine as measured by C_max_ and AUC was found to increase in a dose dependent manner over the dose range of 10–40 mg for both single and multiple dose administration. The safety assessments showed that all treatment related side effects were of mild intensity and there were no serious adverse events (SAEs) or withdrawals due to treatment–emergent adverse events (TEAEs) in this study. The therapeutic dose of rupatadine did not show any CNS impairment in any of the cognitive tests.

**Conclusions:**

This study demonstrated that rupatadine is safe and well tolerated by Japanese healthy subjects. The PK-PD profile confirmed previous experience with rupatadine.

## Introduction

Antihistamines are commonly used as first line treatment to alleviate allergic rhinitis and urticaria. First generation antihistamines were proven to be very effective but have mainly been associated with significant adverse effects on performance and psychomotor activity mediated by their strong H_1_ inhibitory effect [1]. Second-generation antihistamines, with a lower potential for H1-receptor occupancy in the brain, are less likely to produce sedation at recommended dosages [2].

Rupatadine is classified as a new second generation antihistamine that shows affinity for H1-receptor with the advantage of exhibiting additional platelet activating factor (PAF) antagonist activity. The activity have been shown in several *in vitro* and *in vivo* studies and more recently in specific PAF nasal challenge in healthy and allergic rhinitis subjects [3], where rupatadine was the unique treatment able to decrease overall AUC nasal symptoms comparison with placebo. Rupatadine (10 and 20 mg) are effective and well-tolerated for allergic rhinitis [4–6], urticaria [7–11] with no side effects on cardiac repolarization [12] or central nervous system [13].

The pharmacological profile of rupatadine has been described in different dose-ranging trials from 2.5 to 100 mg [12, 14, 15] and an increase of AUC and C_max_ in proportion to the 10–40 mg dose range administered were demonstrated [16]. Rupatadine is almost completely metabolised when administered orally with very little of the drug being recovered unmetabolised [17]. Two of its main metabolites, desloratadine and 3-hydroxylated desloratadine, retain antihistaminic properties which may contribute to the overall efficacy of the drug [14]. Rupatadine is extensively metabolised in the liver and (CYP) 3A4 was identified as the primary isoenzyme responsible for its metabolism [14]. Thus, rupatadine should be used with caution when administered in combination with cytochrome P450 inhibitors, such as erythromycin or ketoconazole. The co-administration of these drugs results in an increased systemic exposure to rupatadine of 10 and 2–3 times for ketoconazole and erythromycin respectively. However, no clinically relevant adverse events were associated with an increased exposure to rupatadine when administered with erythromycin or ketoconazole [14]. Doses up to 100 mg were given to non-Japanese subjects were found to be well tolerated, and safe in terms of cardiac effects, thereby providing a wide therapeutic window [12].

Recently, a study conducted by Xiong et al. indicated that genetic polymorphisms in CYP3A5 and MDR1 encoding P-glycoprotein (P-gp) involved in drug transport and gastrointestinal absorption, may mediate the variability in rupatadine pharmacokinetics in Chinese subjects leading to reduced efficacy [18]. Although it has been suggested that CYP3A5 is an important contributor for the overall CYP3A activities [19], the specificity of CYP3A5 for rupatadine has not been yet fully characterised.

To enable development of the drug it is important to compare the rupatadine pharmacokinetic (PK) and pharmacodynamic (PD) profile in different ethnic groups. Therefore the primary objective of this study was to assess the safety and tolerability of rupatadine following single and multiple oral administrations to healthy Japanese subjects as well. The cardiac safety was evaluated as secondary objective. We have also aimed to investigate the pharmacokinetics of rupatadine and its two main metabolites desloratadine (UR-12790) and 3-hydroxydesloratadine (UR-12788) and pharmacodynamic activity of rupatadine by assessment of dose on cognitive function.

## Methods

The protocol for this trial and supporting CONSORT checklist are available as supporting information; see S1 File and S2 File.

### Ethics Statement

The study protocol (EudraCT: 2012-004900-37) was approved by a National Health Service (NHS) Research Ethics Committee (South Central-Berkshire B, United Kingdom) and the Medicines and Healthcare products Regulatory Authority (MHRA). The study was conducted in accordance with the applicable UK law, the Declaration of Helsinki and Good Clinical Practice guidelines.

### Study Subjects

Eligible subjects were healthy, male or female between the ages of 20 and 45 years, with a body mass index between 18 and 25 kg/m^2^, who were born in Japan to both Japanese parents and grandparents, lived less than 5 years outside of Japan and who did not have significant change in lifestyle, including diet, since leaving Japan. Subjects were judged to be healthy from a medical history, physical examination, routine laboratory investigations, vital signs and 12-lead electrocardiograms (ECGs). Subjects agreed to use an effective method of contraception. Subjects were excluded if they used any substance capable of inhibiting CYP3A4 enzymes within the 2 weeks prior to admission or had any clinical significant disease or any condition that might have affected drug absorption, distribution or excretion. To exclude pregnancy a urinary test was conducted at preening and prior subject’s enrolment. Written informed consent was obtained from each eligible subject prior to the conduct of any study–related procedure.

### Study Design

This was a randomised, double-blind, placebo-controlled trial to determine the safety, tolerability, pharmacokinetics and pharmacodynamics of oral rupatadine in healthy Japanese subjects after single and multiple ascending doses.

The study consisted of three cohorts. Within each cohort, 9 healthy male or female Japanese subjects were randomised to receive the either 10, 20 or 40 mg rupatadine or placebo during 5 days in a 7:2 ratio. Statistical analysis were performed for analysis of variance and comparability of demographic characteristics, among each treatment group.

**Fig 1:**
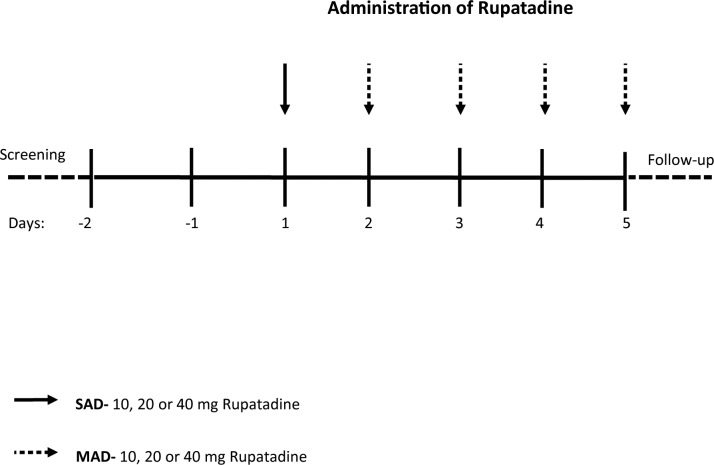
Study design. Schematic representation of the study design. A single dose of rupatadine was given on Day 1 and multiple ascending doses were administered on days 2–5.

The subjects were admitted on Day -2 (i.e. 2 days before the administration of the first dose), received placebo on Day -1 and a single dose of rupatadine or placebo on Day 1 followed by once daily doses on Days 2–5. There was no washout following the single dose on Day 1 as only PK parameters up to 24 h were estimated (Fig 1). On dosing days, standardised meals were served at the following times: breakfast 2 h post-dose, lunch 6 h post-dose, and dinner 12 h post-dose. Water was not permitted from 1 h pre-dose to 4 h post-dose. On Days 6, 7, 8, 9, 10 and 11 after the last dose subjects attended the unit for PK sampling and a general follow-up assessment.

### Pharmacokinetic Assessments

Blood samples were collected on Days 1 and 5 at pre-dose, 0.33, 0.66, 1, 1.5, 2, 3, 4, 6, 8 and 12 h after study drug administration, on Days 2–4 at pre-dose and at 24, 48, 72, 96, 120 and 144 h post-dose. Urine samples were collected on Days 1 and 5 at pre-dose and from 0–24 h after dose administration and on Days 7 (24–48 hours post-dose), 8 (48–72 hours post-dose) and 9 (72–96 hours post-dose). Concentrations of rupatadine and its metabolites UR-12790 (desloratadine) and UR-12788 (3-hydroxydesloratadine) were analysed by Laboratorios Echevarne on behalf of J. Uriach y Compañía, S.A using a validated method of liquid chromatography with tandem mass spectrometry (LC-MS/MS).

Descriptive statistics were used to summarise PK parameters by treatment group. PK parameters were derived by non–compartmental analysis using Phoenix WinNonlin V6.3. Statistical Analysis System (SAS^TM^) v9.2 was used for statistical analysis. PK parameters included: maximum plasma concentration (C_max_), time to C_max_ (t_max_), area under the plasma concentration versus time curve from zero to infinity (AUC_0–∞_), AUC_0-τ_ (where τ = 24 h) and half–life (t_1/2_).

To assess the dose proportionality of PK parameters a linear regression model of the natural log transformed values with the intercept and natural log transformed dose fitted as fixed effects was applied. The dose proportionality was confirmed if the 90% confidence interval (CI) of the slope (β) ranged between 0.8–1.25. Other criteria used were the inclusion of 1 in the 90% CI of the slope (β) and also the proximity to 1 of this estimated parameter.

### Cognitive Function Assessment

Cognitive function was assessed with Cogtest (Cogtest, London, UK), a customized computerized cognitive test battery. The Cogtest Battery in this study included: rapid visual information processing continuous performance task (RVP—CPT Flanker) to assess the subject's ability to sustain attention, reaction time (RT) to assess psychomotor speed, spatial working memory (SWM) to measure the recall of special locations and visual analogue scales (VAS) to assess the level of drowsiness and fatigue. The RVP-CPT flanker test used flanker stimuli. The subjects were expected to respond in terms of correct or incorrect depending on whether the middle element in a display of 5 lines has an arrowhead pointing to the right or left. The middle element is the ‘target’ and the other 4 lines are ‘flankers’. On neutral trials the flankers had no arrowheads as they were just horizontal lines. On congruent trials all flankers had arrowheads pointing in the same direction as the target. On incongruent (conflict) trials, the flankers had arrowheads pointing in the direction opposite that of the target.

Simple reaction time was the time (msec) taken between a stimulus and a movement where the appearance of the stimulus was visual and occurred after a random delay from the presentation of the stimulus. Spatial working memory test was designed to determine how accurately subjects recall the special locations of briefly presented visual targets (pixel). The scale to assess the level of drowsiness and fatigue consists of 8 related items: alert/drowsy; active-passive; mentally slow/quick witted; well-coordinated/clumsy; tense/relaxed; calm/excited; not competent /efficient; attentive/dreamy. The subjects rated each item depending on how she/he were feeling at the time. The battery of tests was chosen on the basis of observed events from a study in non-Japanese subjects investigating the effects of different doses of rupatadine on cognitive function [13].

Site staff members were trained and certified to administer the tests using computers. All subjects attended two training sessions on Day -2 and the cognitive tests were performed on Days -1, 1 and 5 at 1 and 3 h after administration of placebo or rupatadine.

Cognitive tests were summarised by arithmetic and geometric means, standard deviations (SD), minimum, maximum and median values, and coefficients of variation. Each of the cognitive test outcomes differences between treatment groups were assessed by Kruskal-Wallis tests based on changes from time matched baseline (Day -1) [20] in a descriptive way after single and multiple doses.

### Safety and Tolerability

Adverse events (AEs) were recorded from the signing of the informed consent until the end of the study, on Day 11. Standard Toxicity grading according to the National Cancer Institute Common Terminology Criteria for Adverse Events (NCI CTCAE version 4.0) was used to grade the adverse events. The duration, intensity, outcome, and potential relationship with the study drug of each AE were assessed. Safety assessments included standard laboratory safety tests (haematology, biochemistry and urinalysis), vital signs, 12–lead electrocardiogram (ECG), 5-lead Holter ECG, 12-lead telemetry and physical examination. Descriptive statistics were used to summarize the safety data and AE recording.

## Results

### Subjects demographics

**Fig 2:**
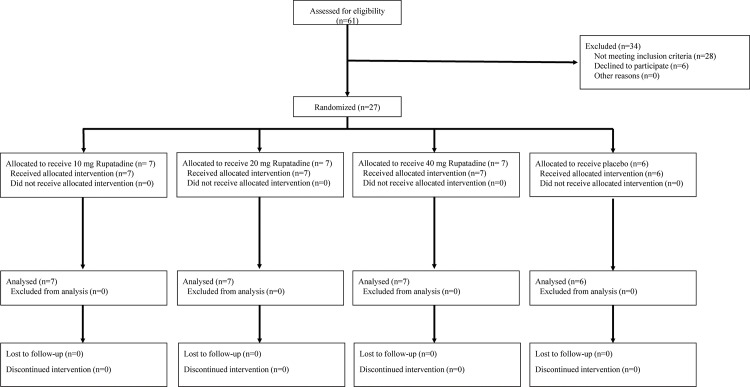
CONSORT 2010 Flow Diagram. Outlined design of the clinical study.

This study was carried out for a period of 15 weeks from the date of first enrolment on 30^th^ November 2012 to the date of last follow-up on 17^th^ March 2013. The CONSORT 2010 flow diagram is shown in Fig 2. Of the 61 subjects assessed for eligibility at Richmond Pharmacology Ltd, 28 did not fulfil the entry criteria of the study and 6 declined to participate. Twenty-seven male and female Japanese subjects were randomised to receive allocated treatment and completed all study assessments. The demographic data for these subjects are summarised in Table 1. The statistical analyses showed no differences among treatment groups.

**Table 1:** Demographic characteristics of treatment groups.

Variable	Rupatadine 10 mg	Rupatadine 20 mg	Rupatadine 40 mg	Placebo	Overall
**n**	7	7	7	6	27
**Age (years)**	28.43±4.20	26.14±4.88	27.00±3.83	29.33±3.27	27.67±4.07
**Gender (n = Male/Female)**	5/2	5/2	3/4	3/3	16/11
**Height (cm)**	169.86±9.63	170.29±5.19	161.43±7.39	168.17±6.62	167.41±7.89
**Weight (kg)**	62.87±11.44	61.57±7.18	57.26±9.22	63.57±3.31	61.23±8.37
**BMI (kg/m**^**2**^**)**	21.67±1.93	21.20±1.79	21.91±2.54	22.52±1.34	21.79±1.91

N: number; BMI: Body Mass Index; N/A: non-applicable

### Pharmacokinetics

**Fig 3:**
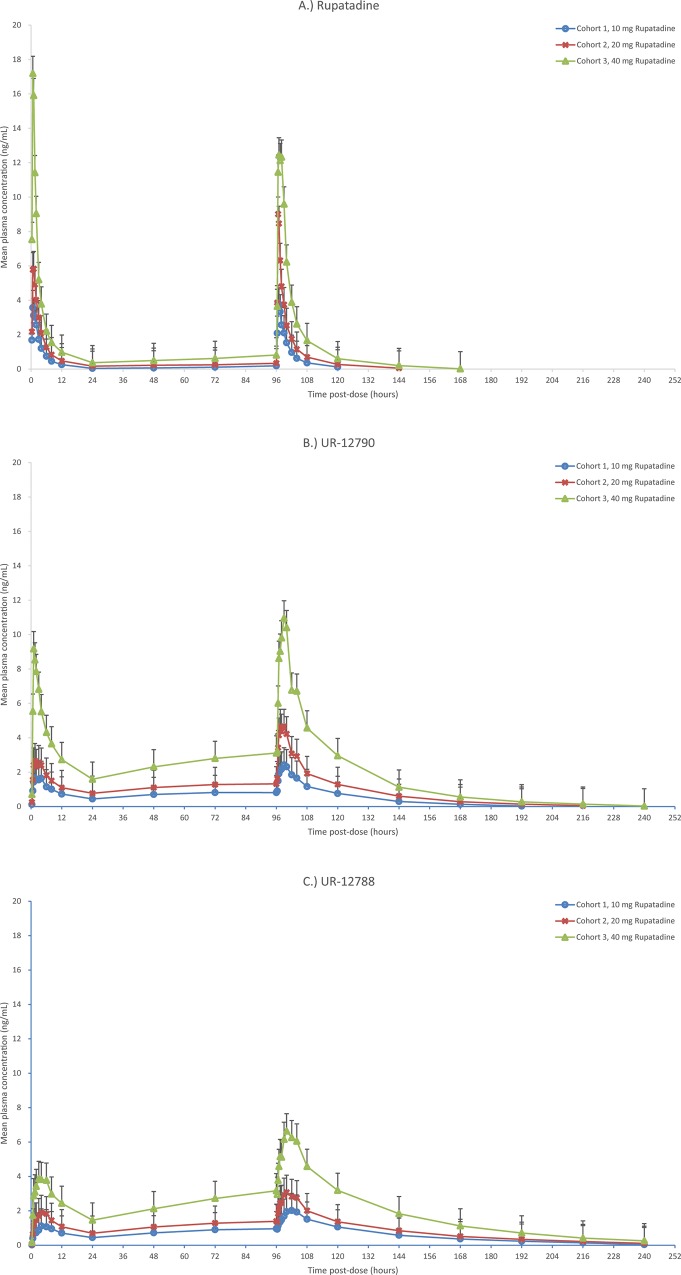
Mean (±SD) plasma concentration. Profiles of rupatadine over time (A), UR-12790 (desloratadine) (B) and UR-12788 (3-hydroxydesloratadine) (C) following administration of single and multiple doses of rupatadine (10, 20 and 40 mg) on Days 1 and 5.

Following single and multiple dosing of rupatadine, the PK data demonstrated that the mean plasma concentrations of rupatadine and its two metabolites UR-12790 (desloratadine) and UR-12788 (3-hydroxydesloratadine) increased with rising dose levels from 10 to 40 mg. The mean plasma concentration profiles over time for rupatadine, UR-12790 and UR-12788 are presented in Fig 3 and the descriptive pharmacokinetic parameters are summarized in Tables 2 and 3.

**Table 2:** Mean pharmacokinetic (±SD) parameters following administration of single doses of rupatadine (10, 20 and 40 mg). Median (min–max) values are presented for t_max_.

Single Doses (Day 1)				
Parameter	Compound	Cohort 1 Rupatadine 10 mg	Cohort 2 Rupatadine 20 mg	Cohort 3 Rupatadine 40 mg
**C**_**max**_ **(ng/mL)**	**Rupatadine**	4.62±1.51	6.88±3.64	18.13±9.91
	**UR-12790**	2.02±0.70	2.95±0.67	9.91±3.73
	**UR-12788**	1.15±0.19	1.97±0.38	3.98±1.06
**t**_**max**_ **(h)**	**Rupatadine**	0.67(0.67–2.00)	1.00(0.67–1.50)	0.67(0.67–1.53)
	**UR-12790**	1.50(1.00–4.00)	1.52(1.00–4.00)	1.00(1.00–2.00)
	**UR-12788**	4.00(4.00–6.00)	4.00(3.00–6.00)	4.00(2.00–8.00)
**AUC**_**0-τ**_ **(h.ng/mL)**	**Rupatadine**	14.81±5.79	25.94±13.41	56.00±20.84
	**UR-12790**	20.59±6.80	32.19±5.80	81.04±26.38
	**UR-12788**	17.02±2.73	27.60±4.91	58.36±17.95
*AUC_**0-∞**_ **(h.ng/mL)**	**Rupatadine**	15.39±6.45	27.82±14.21	60.25±21.53
	**UR-12790**	29.56±9.96	49.52±11.00	117.07±35.37
	**UR-12788**	26.66±5.70	42.90±8.75	92.68±26.11
**t**_**1/2**_ **(h)**	**Rupatadine**	4.76±2.07	7.09±2.00	7.94±1.29
	**UR-12790**	13.94±2.66	15.40±3.22	15.28±7.26
	**UR-12788**	14.86±2.66	15.09±3.06	16.94±4.22

*AUC value with 20% extrapolation and displayed for informative purposes.

UR-12790: desloratadine; UR-12788: 3-hydroxydesloratadine

**Table 3:** Mean pharmacokinetic (±SD) parameters following administration of multiple doses of rupatadine (10, 20 and 40 mg). Median (min–max) values are presented for t_max_.

Multiple Doses (Days 2–5)				
Parameter	Compound	Cohort 1 Rupatadine 10 mg	Cohort 2 Rupatadine 20 mg	Cohort 3 Rupatadine 40 mg
**C**_**max**_ **(ng/mL)**	**Rupatadine**	5.02±2.08	10.65±5.91	18.23±10.83
	**UR-12790**	2.61±0.5171	5.041±1.363	11.65±3.57
	**UR-12788**	2.10±0.32	3.10±0.59	6.76±1.73
**t**_**max**_ **(h)**	**Rupatadine**	1.00(0.67–1.50)	0.70(0.67–1.50)	1.00(0.67–2.00)
	**UR-12790**	3.00(0.67–4.00)	1.50(0.70–3.00)	1.50(1.00–4)
	**UR-12788**	6.00(4.00–8.00)	4.00(1.50–6.00)	4.00(1.00–6.00)
**AUC**_**0-τ**_ **(h.ng/mL)**	**Rupatadine**	18.57±6.24	35.63±15.58	75.48±35.20
	**UR-12790**	32.67±10.36	57.83±16.96	132.33±34.53
	**UR-12788**	35.85±4.00	50.61±11.47	112.96±30.14
**AUC**_**0-∞**_ **(h.ng/mL)**	**Rupatadine**	20.03±6.99	40.59±16.36	88.29±39.17
	**UR-12790**	54.42±16.24	100.91±34.35	217.01±59.35
	**UR-12788**	85.87±11.36	120.97±29.43	260.89±73.73
**t**_**1/2**_ **(h)**	**Rupatadine**	6.56±2.35	10.57±4.73	12.77±2.12
	**UR-12790**	20.65±3.76	24.79±4.68	24.50±3.60
	**UR-12788**	35.91± 6.55	36.01±6.24	32.97±2.95

UR-12790: desloratadine; UR-12788: 3-hydroxydesloratadine

Rupatadine was shown to be rapidly metabolised and t_1/2_ was shown to prolong in a dose dependent manner ranging from 4.46 to 7.94 h after administration of single doses and ranging from 6.56 to 12.77 h after multiple doses. After 5 days of oral administration of different rupatadine doses the half-life values for UR-12790 and UR-12788 ranged from 20.65–24.79 h and 32.97–36.01 h respectively. Once daily multiple doses of rupatadine did not lead to accumulation but desloratadine and 3-hydroxydesloratadine showed an increase in AUC values.

A dose proportionality analysis of rupatadine and the metabolites was performed. The statistical model was a linear regression model of the natural log transformed values with the intercept and natural log transformed dose fitted as fixed effects. Dose proportionality was declared if the 90% confidence interval (CI) of the slope (β) was within the range of 0.8–1.25. Other criteria of dose proportionality (linearity) were the inclusion of 1 in the 90% CI of the slope (β) and also the proximity to 1 of this estimated parameter. Both criteria were met by rupatadine and UR-12790 for C_max_, AUC_0-∞_ on Day 1 and C_max_, AUC_0-τ_ on Day 5.

For UR-12788 the 90% CI of β for C_max_ and AUC_0-∞_ on Day 1 and C_max_ and AUC_0-τ_ on Day 5 fell outside the range of 0.8 to 1.25, however the 90% CI of β for C_max_ and AUC_0-∞_ on Day 1 included 1.The 90% CI of β for UR-12788 C_max_ and AUC_0-τ_ on Day 5 did not include 1. However as the extrapolated AUC_0-∞_ was higher than 20% for UR-12790 and UR-12788, the determination of dose proportionality cannot be conclusively determined on Day 1 for the metabolites.

In both healthy Japanese male and female subjects, the A_e_ (the amount of drug excreted in the urine) for rupatadine could not be determined because the majority of the values were below the LLOQ (0.5 ng/mL) whereas the A_e_ for URincreased in a dose dependent manner (data not shown).

### Cognitive function

**Fig 4:**
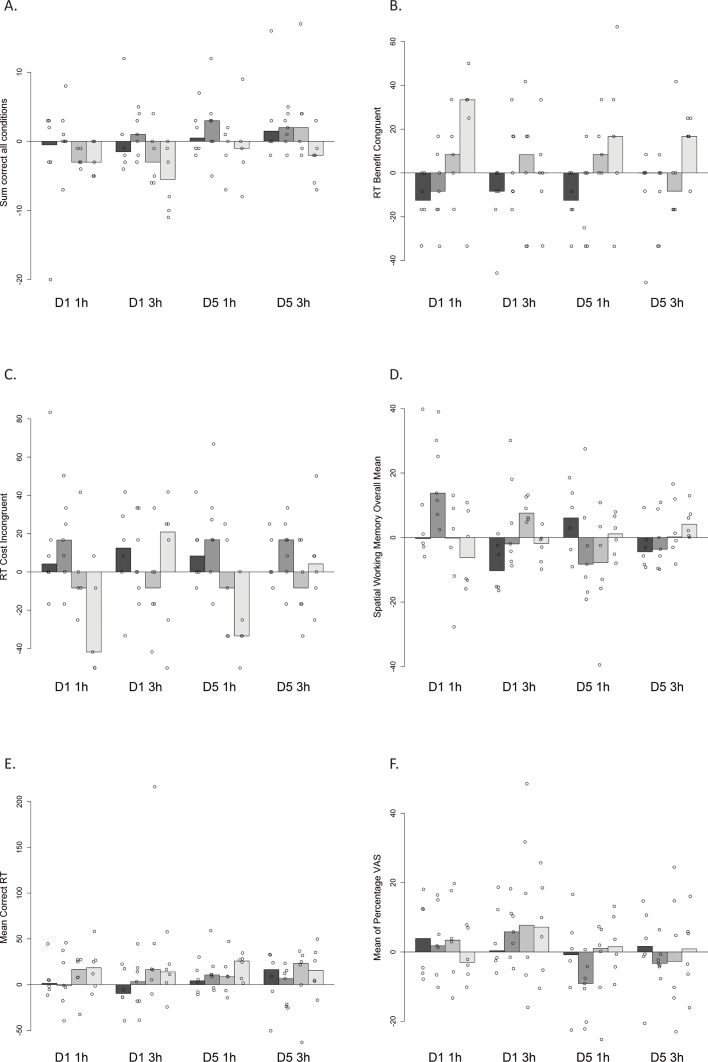
Cognitive function tests. Tests were performed at 1 (t_max_) and 3 h after administration of placebo, 10, 20 and 40 mg of rupatadine (increasing doses from left to right with dark grey representing placebo and lighter grey tones indicate increasing doses) on Day 1 (D1) and Day 5 (D5). A) Rapid Visual Information Processing (sum correct all conditions) test (range– 0–144 msec); B) RT benefit congruent (range -800 +800); C) RT cost incongruent (range -800 +800); D) Spatial Working memory (range 0–1280 pixel); E) Reaction Time (range 200–3000); F) Visual Analogue Scales (range 1–100 percentage). Median values of change from time matched baseline are presented. Individual values are given in addition to the median and represented by white circles.

The effects of rupatadine on sustained attention, reaction time, memory and levels of drowsiness and fatigue are illustrated in Fig 4.

The small sample size in this study allowed us to explore the overall patterns of the data; however the resultant variability precluded significant statistical comparisons between treatment groups using Kruskal-Wallis tests.

The assessment of the cognitive parameters indicated that the therapeutic dose of 10 mg does not present apparent cognitive impairment. Unlike higher doses, rupatadine 10 mg Rapid Visual Processing (RVP) assessment scores were comparable with the placebo treatment group at 1 h of drug administration, the time correspondent to rupatadine t_max_ (Fig 4A). In the same assessment, RVP was shown to be worsened in a dose dependent fashion by higher doses of rupatadine (Fig 4A). Similarly, the RT benefit congruent analysis revealed that compared to placebo, subjects dosed with 20 and 40 mg rupatadine had higher assessment scores at 1h after dosing on Day 1 and Day 5 indicating a poorer cognitive control at higher doses (Fig 4B). In addition, the RT cost incongruent analysis showed that subjects dosed with higher doses of rupatadine had lower assessment scores in comparison to placebo 1 h after administration of rupatadine on Days 1 and 5 (Fig 4C). These findings suggest that the effects on cognitive function appear to be dose dependent and more pronounced at around t_max_ on Days 1 and 5 at high doses with some development of rupatadine tolerance after multiple doses.

Spatial working memory measurements did not suggest a relationship between SWM scores and dose levels (Fig 4D). Subjects in the 20 and 40 mg treatment groups but not in the 10 mg group had prolonged reaction times in comparison with placebo on Days 1 and 5 (Fig 4E). This increase seemed more apparent for the 40 mg dose after 1 h. In addition, no significant effects on the level of drowsiness and fatigue were observed (Fig 4F) as evaluated by visual analogue scales (VAS) apart from some tendency for drowsiness at all doses levels at 3 h on Day 1.

### Safety and tolerability

All subjects on placebo treatment (n = 6) and 21 subjects on rupatadine have received all planned doses. Two of the 27 subjects reported three, treatment emergent adverse events (TEAEs). One TEAE that was judged by the investigator as possibly treatment related occurred after 2 h of administration of 10 mg rupatadine on Day 1. The subject experienced somnolence of mild intensity that lasted 47 h. One subject in the placebo treatment group reported two TEAEs. One instance of mild intensity nausea was reported on Day 1 and swelling inside the mouth was also reported on Day 8. All TEAEs resolved without the use of corrective therapy. There were no TEAEs reported after administration of 20 and 40 mg rupatadine. No clinically significant changes were detected in the laboratory parameters, physical examinations and vital signs. The effects of rupatadine on ECGs were reported elsewhere. There were no serious adverse events (SAEs) during the study and no AEs that lead to subject withdrawal.

## Discussion

Previous studies of the PK, PD and safety profile of rupatadine had only included non-Japanese subjects. This was the first clinical trial showing that rupatadine is safe and well tolerated after single and multiple oral doses administration (10, 20 and 40 mg) in Japanese subjects. The results from this study suggest that there were no significant differences between Japanese and non-Japanese regarding safety, tolerability, PK and PD characteristics of rupatadine when compared with published data.

Cognitive effects of the different rupatadine doses were assessed to exclude large differences between Japanese and non-Japanese subjects. The choice of assessments was based in a previous study showing an evident CNS impairment activity at higher doses (80 mg) in white subjects while therapeutically relevant lower doses were similar to placebo [13]. It was also evidenced, even though with lower magnitude, that subject's attention skills were reduced after single doses of 20 and 40 mg of rupatadine in non-Japanese subjects, simple reaction times were increased in all treatments and effects in activity and drowsiness were obtained with 10, 20 and 40 mg between 1 and 4 h after drug administration [13].

These findings are comparable with the data from the present study. Similarly, no relevant changes in relation to placebo were evidenced after administration of the therapeutic dose of rupatadine (10 mg). Conversely, higher doses of rupatadine (20 and 40 mg) when administered to the Japanese subjects participating in this study, seem to impair reaction time and visual performance with a more pronounced effect when maximum plasma concentrations are achieved showing a correlation between plasma concentration and impaired performance. In addition, the relative less noticeable effect of the higher doses after multiple doses on the ability to sustain attention and level of fatigue and drowsiness may be indicative of a gradual increased tolerance. Nevertheless, it is important to note due to the small sample size the power for conducting cognitive assessments in this study is low and only descriptive exploratory data can be produced.

A direct comparison between the data sets from the TQT study by Donado et al. [12] and this study is presented throughout this discussion section. The TQT published study was considered representative in terms of PK in a non-Japanese clinical trial due to the large sample size, similarity in study design as single and multiple doses of rupatadine and placebo were administrated for 5 days and inclusion of a supratherapeutic dose of 10 times the recommended dose of rupatadine.

Rupatadine safety has been extensively evaluated and the results of a 1-year clinical trial testing for the safety of long term use of 10 mg rupatadine [21] confirmed its tolerability, which was consistent with findings from this study and other shorter-term clinical trials. Clinical research experience with rupatadine and white subjects had generally exhibited a favourable safety and tolerability profile at doses ranging from 10 to 100 mg [7, 12]. Somnolence was found to be the most common adverse reaction reported in white studies following oral administration of rupatadine [22]. After administration of single and multiple doses of 10, 20 and 40 mg of rupatadine, the safety results of this study demonstrated that rupatadine was safe and well tolerated by the Japanese subjects. Notably, under 10 mg rupatadine, somnolence was the only TEAE reported in one subject and classified as possibly related with treatment. However, 10 mg rupatadine was not associated with cognitive impairment as described below and reported levels of adverse events were found to be lower in Japanese—a common cultural variation feature in bridging studies [23].

Rupatadine PK parameters in Japanese subjects are shown to be in close agreement with the results exhibited by several white studies [24–26]. Similarly to the linear increase observed in white studies with single doses of 10–40 mg rupatadine [14], dose proportionality analysis in this study revealed that the estimated slope values for C_max,_ AUC_0-∞_ and AUC_0-τ_ after single and repeated doses of rupatadine supports linearity. The 90% CI of the slope (β) for log transformed C_max_ and AUC_0-τ_ values for the metabolite UR-12788 did not fulfil the criteria of dose proportionality on Day 5. As the 90% CIs of β were compatible with dose proportionality on Day 1, sample size and data variability were potentially the reasons for UR-12788 not meeting dose proportionality criteria following multiple doses of rupatadine suggesting that lower levels of UR-12788 are obtained with higher doses of rupatadine.

Regarding median t_max_ and t_1/2_ in Japanese subjects, rupatadine, UR-12790 and UR-12788 values following oral administration of multiple doses of 10 mg rupatadine were comparable to the values obtained in white [27, 28], suggesting that no differences in the velocity of absorption and rupatadine elimination are expected.

Rupatadine is metabolised in the liver mainly by CYP3A4 through oxidative reactions and eliminated via the bile with negligible amounts of unchanged drug detected in urine as confirmed in this study. It is not known if genetic polymorphisms of CYP3A4 have an effect on rupatadine pharmacokinetics. The impact of CYP3A5 gene polymorphism on the metabolism of rupatadine was investigated in Chinese subjects [18]. In this study it was reported that CYP3A5*1 carriers will have a higher metabolic activity for rupatadine. However, the underlying metabolism of rupatadine was not further explored as the concentrations of metabolites were not measured.

CYP3A5 may be one of the genetic contributors to inter-individual differences in CYP3A-dependent drug metabolism. Some ethnicities such as Chinese were shown to have a high prevalence (40–60%) of CYP3A5 expression [19]. Among white and Japanese the polymorphic distribution of CYP3A5*1 indicates that 30% of both populations may metabolize CYP3A substrates more rapidly [19] which suggest that rupatadine biotransformation is expected to be consistent between these two ethnic groups. The individual pharmacokinetic parameters of subjects enrolled in this study and the study conducted by Donado et al. [12] showed no significant differences in the exposure profiles of rupatadine 10 mg and its metabolites supporting a similar metabolic rate for rupatadine within these populations (data not shown). Additionally, polymorphism analysis performed on subjects enrolled in this study (data not shown) did not support the Xiong et al findings CYP3A5 genotype was not correlated with differences in rupatadine metabolism and pharmacokinetics.

Slow metaboliser phenotypes have also been identified in the metabolism of the rupatadine primary metabolite desloratadine [29, 30] with a prevalence frequency of 17% in blacks, 2% in whites and 2% in Hispanics [31]. A multiple-dose clinical study has demonstrated that rupatadine plasma values for 2 of the 24 subjects enrolled, were within the range observed for other subjects, the corresponding desloratadine levels were high and 3-hydroxyloratadine levels were very low [28]. Subjects with an AUC ratio of 3-hydroxydesloratadine to desloratadine <0.1, or with a desloratadine t_1/2_ >50 h were defined as slow desloratadine metabolisers [28]. These criteria were not fulfilled by any of the subjects enrolled in the present study or the study conducted by Donado et al. [12] (data not shown), i.e. no slow metabolisers for desloratadine were identified in the two studies.

Data from the above trials described suggest that variability in the metabolic pathway of rupatadine can occur. However, from a therapeutic perspective these observations are unlikely to compromise rupatadine safety. The large experience with rupatadine indicates that a vast dose range of rupatadine is safe and well tolerated and increases in bioavailability and exposure to rupatadine associated with concomitant intake of food or cytochrome P450 inhibitors have low clinical relevance regarding safety [14, 27].

In conclusion, this study shows that rupatadine is equally well tolerated by Japanese and non-Japanese subjects after single and multiple oral dose administration (10, 20 and 40 mg). No clinical relevant differences in pharmacokinetic parameters and cognitive function between the two ethnic groups were found supporting the use of rupatadine in Japanese patients with allergic rhinitis and urticaria.

## Supporting Information

S1 File Checklist. (DOC)

S2 File Protocol. (PDF)
