# Supplementary material for: Pharmacokinetics, Safety and Cognitive Function Profile of Rupatadine 10, 20 and 40 mg in Healthy Japanese Subjects: A Randomised Placebo-Controlled Trial
Source: PLoS One. 2016 Sep 15;11(9):e0163020. doi: 10.1371/journal.pone.0163020 (PMC5025135; doi:10.1371/journal.pone.0163020)
Supplement: S2 File — (PDF) [file pone.0163020.s002.pdf]

Clinical Trial Protocol Amendment [Non-Substantial] 1

Sponsor's Reference Number: DC05/RUP/I/13

Richmond Pharmacology Study Number: C11050

EudraCT Number: 2012-004900-37

TITLE: A Phase I, Randomised, Double-blind, Placebo-controlled, Parallel group study to assess the Safety, Tolerability, Pharmacokinetics and Pharmacodynamics of oral Rupatadine in Healthy Japanese Subjects after Single and Multiple Ascending Doses

SPONSOR: J. Uriach y Compañía, S.A.
Avinguda Camí Reial, 51-57
08184 Palau-solità i Plegamans
Barcelona, Spain
Telephone:+34 938632445 Fax: +34 938630310

PRINCIPAL INVESTIGATOR: Dr Jörg Täubel, MD FFPM
Richmond Pharmacology Ltd.
St George's University of London
Cranmer Terrace, Tooting
London SW17 0RE
Telephone: +44 (0)20 8664 5200 Fax: +44 (0)20 8664 5201

Amendment Date: 27 November 2012

Information in this protocol amendment is confidential and should not be disclosed, other than to those directly involved in the execution or the ethical/regulatory review of the trial, without written authorisation from Uriach or its affiliates.

Amendment [Non-Substantial] 1 Signature Page

Protocol No: DC05/RUP/I/13:

A Phase I, Randomised, Double-blind, Placebo-controlled, Parallel group study to assess the Safety, Tolerability, Pharmacokinetics and Pharmacodynamics of oral Rupatadine in Healthy Japanese Subjects after Single and Multiple Ascending Doses

---

This Clinical Trial Protocol and Amendment have been subjected to an internal Uriach peer review.

I agree to the terms of this trial protocol amendment.

Sponsor's Signatory:

Dr Iñaki Izquierdo, MD PhD
Head of Clinical Development and Medical Advice Department

Signature: ______________________ **Date:** 30. NOV. 2012

This Clinical Trial Protocol and Amendment have been subjected to an internal RPL review.

I agree to the terms of this protocol and amendment (Number 1, dated 26 November 2012). I will conduct the trial according to the procedures specified herein, and according to the principles of Good Clinical Practice and local regulations.

Principal Investigator:

Dr Jorg Taubel, MD FFPM

Signature: ______________________ **Date:** 06 Dec 2012

Reason for Amendment:

This non substantial amendment to the protocol has been initiated to change the protocol reference in the header of the entire document, to update the Introduction section to include the use of Ruapatadine in children, to revise the exclusion criteria no 6 and 10 for admission on Day-2, to change the screening holter equipement to 12-lead Holter and aliquots for urine collection. The changes in this non-substantial amendment will be applicable from the first cohort onwards, until further notice, i.e. if there is no further non-substantial amendment the changes will be applicable for subsequent cohorts. If there are additional changes, a new non-substantial amendment will be written. This non-substantial amendment does not require submission to either Medicines and Healthcare products Regulatory Agency (MHRA) or Research Ethics Committee (REC).

Persons who initiated the Amendment:

J. Uriach y Compañía, S.A and Richmond Pharmacology Ltd.

Sponsor:

J. Uriach y Compañía, S.A., Avinguda Camí Reial, 51-57, 08184 Palau-solità i Plegamans, Barcelona, Spain

Centres affected by the Amendment:

Richmond Pharmacology Ltd, UK.

This amendment to the protocol is non-substantial and the changes are given as strikethroughs and underlines.

Section of protocol affected:

1) Header of entire document
2) Section 6.0 Introduction, page 13
3) Section 9.3 Exclusion Criteria 6 and 10, page 31
4) Section 12.5, Holter ECG Recordings, page 38
5) Section 12.10.2 Urine Collection, page 41

1) **Header of Entire document**

Previous text:

J. Uriach y Compañía, S.A.
Protocol Ref: DC05/RUP/I/133

RPL Study No. C11050

Revised text:

J. Uriach y Compañía, S.A.
Protocol Ref: DC05/RUP/I/13~~3~~
RPL Study No. C11050

2) Section 6.0 - Introduction page 13

Previous text:

Rupatadine is a marketed drug indicated for symptomatic treatment of allergic rhinitis and urticaria in adults and adolescents.

Revised text:

Rupatadine is a marketed drug indicated for symptomatic treatment of allergic rhinitis and urticaria in adults and adolescents <u>and was recently also approved in Europe for children aged 6-11 years for allergic rhinitis.</u>

3) Section 9.3 - Exclusion Criteria 6 and 10 page 31

Previous text: Confirmed positive results from urine drug screen (amphetamines, benzodiazepines, cocaine, cannabinoids, opiates, barbiturates, and methadone) or from the alcohol breath test at screening and on admission (Day -1).

Use of any medication (including over-the-counter (OTC) medication) within 2 weeks prior to admission (Day -1) or within less than 10 times the elimination half-life of the respective drug, or anticipated concomitant medication during the treatment periods. Single intake of a drug may be accepted if judged by the investigators to have no clinical relevance and no relevance for the trial objectives.

Revised text:

Confirmed positive results from urine drug screen (amphetamines, benzodiazepines, cocaine, cannabinoids, opiates, barbiturates, and methadone) or from the alcohol breath test at screening and on admission (Day ~~-1~~ <u>-2</u>).

Use of any medication (including over-the-counter (OTC) medication) within 2 weeks prior to admission (Day ~~-1~~ <u>-2</u>) or within less than 10 times the elimination half-life of the respective drug, or anticipated concomitant medication during the treatment periods. Single intake of a drug may be accepted if judged by the investigators to have no clinical relevance and no relevance for the trial objectives.

4) Section 12.5 page 38

Previous text:

Holter ECG Recordings

- Holter recording will be performed at screening as described in the study plan (Table 2) using a Zymed DigiTrack Plus® Digital Holter Recorder, a compact Holter ambulatory ECG device that records and stores continuous ECGs. After the recording is finished and the device detached from the subject, the data will be downloaded and analysed using Philips 2010 Plus software. The Holter reports will be reviewed and signed off by a qualified cardiologist. In case they are available at screening, Holter reports will be valid if no older than 3 months. The screening Holter recordings will not be entered into the data base.

Revised text:

- Holter recording will be performed at screening as described in the study plan (Table 2) using a ~~Zymed DigiTrack Plus® Digital Holter Recorder~~ GE Getemed 12 Lead Holter, a compact Holter ambulatory ECG device that records and stores continuous ECGs. After the recording is finished and the device detached from the subject, the data will be downloaded and analysed ~~using Philips 2010 Plus software~~. The Holter reports will be reviewed and signed off by a qualified cardiologist. In case they are available at screening, Holter reports will be valid if no older than 3 months. The screening Holter recordings will not be entered into the data base.

5) Section 12.10.2

Previous text:

Urine Collection

Urine samples (Two aliquots of approximately 3 mL each i.e. 6 mL in total, in polypropylene tubes) for determination of concentration of Rupatadine and its two main metabolites UR 12790 and UR 12788 in urine will be taken from the total urine sample provided during each collection period presented in the study plan (Table 2). With the exception of the 0 hour (pre-dose) sample, total volume assessed by weight (g) of each urine collection interval will be recorded. Samples will be collected, stored and shipped as detailed in a separate SOM.

Revised text:

Urine samples (~~Two~~ Three aliquots of approximately ~~3~~ 2 mL each i.e. 6 mL in total, in polypropylene tubes) for determination of concentration of Rupatadine and its two main metabolites UR 12790 and UR 12788 in urine will be taken from the total urine sample provided during each collection period presented in the study plan (Table 2). With the exception of the 0 hour (pre-dose) sample, total

volume assessed by weight (g) of each urine collection interval will be recorded. Samples will be collected, stored and shipped as detailed in a separate SOM.

CLINICAL STUDY PROTOCOL

Sponsor's Reference Number: DC05/RUP/I/13

Richmond Pharmacology Study Number: C11050

EudraCT Number: 2012-004900-37

**TITLE:**	A Phase I, Randomised, Double-blind, Placebo-controlled, Parallel group study to assess the Safety, Tolerability, Pharmacokinetics and Pharmacodynamics of oral Rupatadine in Healthy Japanese Subjects after Single and Multiple Ascending Doses
**PHASE:**	Phase I
**DRUG:**	Rupatadine
**SPONSOR:**	J. Uriach y Compañía, S.A.<br>Avinguda Camí Reial, 51-57<br>08184 Palau-solità i Plegamans<br>Barcelona, Spain<br><br>Telephone:+34 938632445 Fax: +34 938630310
**PRINCIPAL INVESTIGATOR:**	Dr Jorg Taubel, MD FFPM<br>Richmond Pharmacology Ltd.<br>St George's University of London<br>Cranmer Terrace,<br>London SW17 0RE, UK<br>Telephone: +44 (0)20 8664 5200 Fax: +44 (0)20 8664 5201
**STUDY SITE:**	Richmond Pharmacology Ltd.<br>Croydon University Hospital<br>Woodcroft Wing<br>530 London Road<br>Croydon<br>Surrey CR7 7YE, UK
**Protocol Version and Date:**	Version 3.0<br>02 November 2012

Information in this protocol is confidential and should not be disclosed, other than to those directly involved in the execution or the ethical/regulatory review of the study, without written authorisation from J. Uriach y Compañía, S.A. or its affiliates.

1. PROTOCOL APPROVAL SIGNATURES

Protocol Version 3.0, dated 02 NOV 2012

Sponsor's Approval

This protocol has been approved by J. Uriach y Compañía, S.A.

Sponsor's Signatory:
Dr Iñaki Izquierdo, MD PhD
Head of Clinical Development and Medical Advice Department

Signature: ______________________ **Date:** 5. NOV. 2012

Investigator's Agreement

I have read this J. Uriach y Compañía, S.A. Protocol No. DC05/RUP/I/13:

A Phase I, Randomised, Double-blind, Placebo-controlled, Parallel group study to assess the Safety, Tolerability, Pharmacokinetics and Pharmacodynamics of oral Rupatadine in Healthy Japanese Subjects after Single and Multiple Ascending Doses

I have fully discussed the objectives of this trial and the contents of this protocol with the Sponsor's representatives.

I understand that the information in this protocol is confidential and should not be disclosed, other than to those directly involved in the execution or the ethical/regulatory review of the trial, without written authorisation from J. Uriach y Compañía, S.A.. It is, however, permissible to provide information to a subject in order to obtain consent.

I agree to conduct this trial according to this protocol and to comply with its requirements, subject to ethical and safety considerations and guidelines, and to conduct the trial in accordance with the International Conference on Harmonisation (ICH) guidelines on Good Clinical Practice (GCP) and with the applicable regulatory requirements.

I understand that J. Uriach y Compañía, S.A. may decide to suspend or prematurely terminate the trial at any time for whatever reason; such a decision will be communicated to me in writing. Conversely, should I decide to withdraw from execution of the trial I will communicate my intention immediately in writing to J. Uriach y Compañía, S.A.

Principal Investigator:
Dr Jorg Taubel, MD FFPM

Signature: ______________________ **Date:** 02 NOV 2012

2. STUDY PERSONNEL

Sponsor's Medical Contact	Dr Iñaki Izquierdo, MD PhD<br>Head of Clinical Development and Medical Advice Department<br>J. Uriach y Compañía, S.A.<br>Avinguda Camí Reial, 51-57<br>08184 Palau-solità i Plegamans<br>Barcelona, Spain
Sponsor's Clinical Trial Manager	Eva Santamaria Masdeu<br>Clinical Trial Manager<br>J. Uriach y Compañía, S.A.
Principal Investigator	Dr Jorg Taubel, MD FFPM<br>Richmond Pharmacology Ltd.<br>St George's University of London<br>Cranmer Terrace<br>London SW17 0RE, UK<br>Telephone: +44 (0)20 8664 5200 Fax:+44 (0)20 8664 5201<br>e-mail: j.taubel@richmondpharmacology.com
Co-investigator(s)	Dr Ulrike Lorch, MD FRCA FFPM<br>Dr Radivoj Arezina, MD MSc<br>Richmond Pharmacology Ltd
Study Site	Richmond Pharmacology Ltd.<br>Croydon University Hospital<br>Woodcroft Wing<br>530 London Road<br>Croydon<br>Surrey CR7 7YE, UK
Clinical Laboratory	The Doctor's Laboratory<br>60 Whitfield Street<br>London W1T 4EU, UK
Analytical Laboratory (PK)	Laboratorios Echevarne
Statistics	Richmond Pharmacology Ltd.
Data Management	Richmond Pharmacology Ltd.
Medical Writing	Richmond Pharmacology Ltd.

3. TABLE OF CONTENTS

1. PROTOCOL APPROVAL SIGNATURES 2

2. STUDY PERSONNEL 3

3. TABLE OF CONTENTS 4

4. LIST OF ABBREVIATIONS 8

5. STUDY SYNOPSIS 9

6. INTRODUCTION 13

6.1 Pharmacokinetic properties 13
6.1.1 Absorption and bioavailability 13
6.1.2 Effect of the intake of food 13
6.1.3 Metabolism and elimination 14
6.2 Undesirable effects 14
6.3 Rationale for conducting study 15
6.4 Risk/benefit and ethical assessment 15
6.4.1 Potential Benefits 15
6.4.2 Potential Risks 15

7. STUDY OBJECTIVES AND RATIONALE 15

7.1 Study Objectives 15

8. STUDY DESIGN 15

8.1 Overall study design and procedure 15
8.2 Dose escalation 22
8.2.1 Dose escalation procedures 22
8.2.2 Safety Review Committee 22
8.2.3 Stopping criteria 23
8.3 Rationale for study design, doses and control groups 27
8.3.1 Choice of subjects for study 28
8.3.2 Route and rate of administration 28
8.3.3 Estimation of first dose in human subjects 28
8.3.4 Precautions to be applied for dosing between subjects within a cohort 28
8.3.5 Precautions to be applied for dosing between different cohorts 28
8.3.6 Dose escalation scheme 28
8.3.7 Stopping criteria 28
8.3.8 Monitoring and communication of adverse events / reactions 28
8.3.9 Investigator site facilities and personnel 29

9. SELECTION AND WITHDRAWAL OF SUBJECTS 29

9.1 Number and Source of Subjects 29
9.2 Inclusion Criteria 29

9.3 Exclusion Criteria 31
9.4 Subject Restrictions 32
9.5 Withdrawal of Subjects 33
9.5.1 Criteria for withdrawal 33
9.5.2 Procedures for subject withdrawal 33

10. STUDY AND CONCOMITANT TREATMENTS 34
10.1 Investigational Medicinal Products (IMPs) 34
10.2 Doses and treatment regimens 34
10.3 Packaging and Labelling of IMPs 34
10.4 Drug Accountability 35
10.5 Randomisation 35
10.6 Allocation of Subject Numbers 35
10.7 Blinding and Procedures for Unblinding the Study 35
10.7.1 Methods for ensuring blinding 35
10.7.2 Methods for unblinding the study 36
10.8 Concomitant Medications 36

11. STUDY PROCEDURES 36
11.1 Schedule of Study Procedures 36

12. STUDY METHODOLGY 36
12.1 Meals 36
12.2 Vital Signs 37
12.3 Standard 12-lead ECG Recordings 37
12.3.1 Cardiologist over-reading of standard 12-lead ECGs 37
12.4 Telemetry ECG Recordings 38
12.5 Holter ECG Recordings 38
12.6 Physical Examination, Height and Weight 38
12.7 Safety Laboratory Tests 38
12.7.1 Haematology and biochemistry 38
12.7.2 Serology 39
12.7.3 Urinalysis 39
12.7.4 Pregnancy test 39
12.7.5 Drugs of Abuse 39
12.8 Alcohol Breath Test 40
12.9 Cognitive Testing 40
12.10 Pharmacokinetic (PK) Assessments 41
12.10.1 Collection of PK Samples 41
12.10.2 Urine Collection 41
12.10.3 Determination of Drug Concentrations in PK samples 41
12.11 Exploratory Sample 41
12.12 Volume of Blood Sampling 41

13. ADVERSE EVENTS 42

13.1 Adverse Events 42
13.1.1 Definitions 42
13.1.2 Recording of adverse events 44
13.1.3 Assessment of adverse events 44
13.1.4 Reporting of serious adverse events 46
14. QUALITY ASSURANCE AND QUALITY CONTROL 47
14.1 Monitoring 48
15. STATISTICAL EVALUATION 48
15.1 Statistical Analysis Plan 48
15.2 Analysis Sets 48
15.2.1 Safety set 48
15.2.2 PK set 48
15.2.3 PD set 49
15.3 PK Parameters 49
15.4 PD Parameters 51
15.5 Safety Parameters 51
15.6 Statistical analysis 51
15.6.1 PK Analysis 51
15.6.2 PD Analysis 51
15.6.3 Safety Data Analysis 52
15.7 Handling of Missing and Incomplete Data 52
15.8 Sample Size Considerations 53
16. DATA MANAGEMENT 53
16.1 Case Report Forms 53
17. SPONSOR'S AND INVESTIGATOR'S RESPONSIBILITIES 54
17.1 Sponsor's Responsibilities 54
17.1.1 GCP compliance 54
17.1.2 Regulatory approval 54
17.1.3 Protocol management 54
17.1.4 End of trial notification 54
17.1.5 Submission of summary of clinical trial report to competent authorities of member states concerned and RECs 54
17.2 Investigator's Responsibilities 55
17.2.1 GCP compliance 55
17.2.2 Regulatory approval 55
17.2.3 Indemnity/liability and insurance 55
17.2.4 Protocol adherence and investigator agreement 55
17.2.5 Pharmacovigilance 55
17.2.6 Documentation and retention of records 56
17.3 Ethical considerations 56
17.3.1 Informed consent 56

17.3.2 Research Ethics Committee (REC) approval 56
17.4 Confidentiality 57
17.5 Publication Policy 57

18. REFERENCES 59

List of Tables

Table 1 Adaptive protocol features 17
Table 2 Study Plan 20
Table 3 Assessment Schedule 21
Table 4 Group stopping criteria based on clinically significant drug-related adverse events 25
Table 5 Individual stopping criteria based on clinically significant drug related adverse events 26
Table 6 Safety Laboratory Parameters 40
Table 7 Volume of Blood to be Drawn from Each Subject 42

4. LIST OF ABBREVIATIONS

Abbreviation	Explanation
ABPI	Association of British Pharmaceutical Industry
AE	Adverse event
AUC	Area under the plasma concentration versus time curve
BMI	Body mass index
BP	Blood pressure
C_{max}	The observed maximum plasma concentration
CPU	Clinical Pharmacology Unit
CRF	Case Report Form
DCF	Data Clarification Form
DHP	Data handling protocol
ECG	Electrocardiogram
EU	European Union
GCP	Good Clinical Practice
HIV	Human Immunodeficiency Virus
HR	Heart rate
ICH	International Conference on Harmonisation
IMP	Investigational Medicinal Products
LLOQ	Lower Limit of Quantification
MedDRA	Medical Dictionary for Regulatory Activities
NCI CTCAE	National Cancer Institute Common Terminology Criteria for Adverse Events
PD	Pharmacodynamic
PK	Pharmacokinetic
PMDA	Pharmaceuticals and Medical Devices Agency
QC	Quality Control
REC	Research Ethics Committee
RPL	Richmond Pharmacology Ltd
SAE	Serious adverse event
SAS	Statistical Analysis System
SmPC	Summary of Product Characteristics
SOM	Study Operations Manual
SRC	Safety Review Committee
SUSAR	Suspected Unexpected Serious Adverse Reactions
WHO	World Health Organisation

5. STUDY SYNOPSIS

**Protocol Ref.** DC05/RUP/I/13	**Study drug:** Rupatadine

Title of the study:

A Phase I, Randomised, Double-blind, Placebo-controlled, Parallel group study to assess the Safety, Tolerability, Pharmacokinetics and Pharmacodynamics of oral Rupatadine in Healthy Japanese Subjects after Single and Multiple Ascending Doses

Principal Investigator:

Dr Jorg Taubel, MD FFPM

Study centre:
Richmond Pharmacology Ltd, Croydon University Hospital, Woodcroft Wing, 530 London Rd, Croydon, CR7 7YE (clinic)

Richmond Pharmacology Ltd, St George's University of London, Cranmer Terrace, London, SW17 0RE (cardiological services).

**Study period (planned):** December 2012 (first subject first dose) – March 2013 (last subject last visit)	**Clinical phase:** I

Objectives:

Primary

1. To assess the safety and tolerability of RUPATADINE following single and multiple oral administrations to healthy Japanese subjects.

Secondary

1. To investigate the pharmacokinetics (PK) of RUPATADINE and its two main metabolites desloratadine (UR 12790) and hydroxylatedesloratadine (UR 12788) in plasma and urine (including free and conjugated UR12788) following single and multiple dosing of RUPATADINE once daily in healthy Japanese subjects.
2. To investigate the pharmacodynamic (PD) activity of RUPATADINE by assessment of dose on cognitive function.
3. To measure the effect of RUPATADINE on ECG parameters.

Study Design:

This will be a single centre, randomised, placebo-controlled parallel group study.

The study will be conducted in three cohorts of subjects dosed with Rupatadine or matching placebo. All subjects will receive placebo on Day -1. A single dose of Rupatadine or placebo will be administered on Day 1 followed by once daily doses on Days 2-5.There will be no washout following the single dose on Day 1 as only PK parameters up to 24 hours will be estimated after a single dose.

Since Rupatadine is a marketed drug with a very good safety profile, there is no requirement to stagger dosing within each cohort (sentinel groups). Rupatadine was well tolerated in a clinical safety study at daily dose of 100 mg over 5 days. The starting dose of Rupatadine in this trial will be the normal therapeutic dose of 10mg. The other two anticipated dose levels are 20 mg and 30 mg. After the first and second cohort, a Safety Review Committee (SRC) will decide the dose for the next dose level, based on all cumulative data to date. For progressing to the second dose level, safety and tolerability data will be required; for progressing to the third dose level, safety tolerability and PK data will be required. If significant differences in pharmacokinetics are seen in Japanese subjects compared to the historical PK data in Caucasians, the dose for the third dose level will be decided as appropriate by the SRC. In any event, the highest dose will not exceed 100 mg Rupatadine OR a dose that is expected to yield plasma levels of Rupatadine and/or its metabolites which will not go above the levels seen in Caucasian volunteers after a 100 mg dose. The SRC may decide to hold additional SRC meetings at any time during the trial.

General eligibility of subjects for participation in this study will be assessed at screening which will take place within 21 days of the first study drug administration. Subjects will be resident from the night before the baseline assessment day (Day -2) until 96 hours after their last dose (Day 9). They will attend the unit on Days 10 and 11 (at 120 and 144 hours after last dose); their last visit will also serve as the post study follow up.

Number of subjects:

Twenty seven eligible subjects will be randomised to one of the two treatments. It is anticipated that 21 subjects

will be randomised to receive single daily oral doses of Rupatadine 10 mg, 20 mg and 30 mg; (seven subjects per dose level) and that six subjects will receive matching placebo (two per dose level). Additional subjects may be randomised to ensure that the target of obtaining Rupatadine PK, safety and tolerability data from a minimum of 6 subjects per dose level has been met. Additional subjects may be joined to a later cohort as is deemed practicable by the PI.

Diagnosis and main criteria for admission:

Subjects will be included if they are male or female, Japanese, 20-45 years (inclusive) of age, with a body mass index of 18 to 25 kg/m² inclusive, using an effective contraceptive method (or are abstinent), judged to be healthy from a medical history, physical examination, safety laboratory investigations and screening electrocardiogram (ECG).

Main exclusion criteria are: any pathology or abnormality which is judged to bear a greater than minimal risk for the volunteers to participate and any factor jeopardising the scientific outcome of the trial, the use of concomitant medications, any current psychiatric disorder and inability to give informed consent. All subjects included in the study must meet the ECG screening selection criteria. ECG criteria to be signed off for inclusion by a cardiologist (if applicable).

Test treatment(s) and mode of administration

Rupatadine 10 mg tablets will be given orally after an overnight fast with 240 mL of filtered tap water: anticipated doses are 10 mg for group 1(one tablet), 20 mg for group 2 (two tablets) and 30 mg (three tablets) for group 3.

Matching Placebo tablets will be given orally after an overnight fast with 240 mL of filtered tap water: anticipated doses are one tablet for group 1, two tablets for group 2 and three tablets for group 3.

Duration of treatment:

Single administration of Placebo on Day -1 in the baseline part

Single administration of treatments (Rupatadine or Placebo) on Day 1 in the SAD part

Four daily administrations on Days 2-5 in the MAD part

Key Assessments (for other tests refer to Table 2):

Pharmacokinetic analysis

Blood sample taken through an indwelling venous catheter or venipuncture and urine collections will be performed. Details specified in Study Operations Manual (SOM).

Blood sampling schedule:

Days 1: pre-dose, 0:20, 0:40, 1:00, 1:30, 2, 3, 4, 6, 8 and 12 hours

Days 2-4: pre-dose

Day 5: pre-dose, 0:20, 0:40, 1:00, 1:30, 2, 3, 4, 6, 8 and 12 hours

Days 6-11: 24, 48, 72, 96, 120 and 144 hours

Pharmacodynamic (Cognitive) analysis

The following cognitive assessments will be performed:

- Rapid Visual Information Processing (RVP)
- Reaction Time (RTI)
- Spatial Working Memory (SWM)
- Visual Analogue Scales (VAS)

Sampling schedule:

Day-2: minimum of two training sessions

Day-1, 1 and 5: 1 hour and 3 hours post dose

ECG analysis

Triplicate 12-lead ECG taken in a supine position after 10 minutes rest at a stable heart rate and preceding the blood sampling. Details will be specified in SOM. Sampling schedule:

Day, 1 and 5: taken at PK sampling time-points *minus 5 minutes* and at 5 hour post-dose (when no PK is taken); Day -1: taken at times equivalent to Day 1 and 5.

12-lead telemetry will be recorded from approximately one hour pre-dose until 6 hours post dose on Day -1, Day 1 and Day 5. The traces will be reviewed for safety only.

Safety analysis

Safety assessments will include standard laboratory safety tests (haematology, biochemistry and urinalysis), vital signs (blood pressure [BP], heart rate, body temperature), physical examination, 12-lead ECG and adverse events (AE) monitoring.

Statistical Methods:

Pharmacokinetic analysis

PK parameters will be derived from the relevant plasma concentration data of Rupatadine and its two main metabolites UR 12790 and UR 12788, by non-compartmental analysis using Statistical Analysis System (SAS^{TM}) v9.2 or above.

Single dose pharmacokinetics: The following PK parameters will be derived to assess the single dose PK and the overall exposure to Rupatadine and the two main metabolites, UR 12790 and UR 12788:

Plasma:

- Maximum plasma concentration (C_{max})
- Time to reach maximum plasma concentration (T_{max})
- Area under the plasma concentration vs time curve from zero to 24 hours post last dose (AUC_{τ}, where τ = 24h post dose on Day 1)
- Area under the plasma concentration vs time curve from zero to infinity ($AUC_{0-\infty}$)
- Half-life ($t_{½}$)
- Apparent volume of distribution (Vz/F) - for Rupatadine only
- Oral plasma clearance (CL/F) - for Rupatadine only

Urine:

- Amount of drug excreted in the urine (Ae)
- Renal clearance (CLR)

Trough plasma concentrations: Pre-dose plasma samples will be taken on Days: 2 (the same sample as 24 hours post dosing on Day 1), 3 and 4 in order to determine the minimum plasma concentration (C_{min}) for Rupatadine and its two main metabolites, UR 12790 and UR 12788.

Steady state pharmacokinetics: The following PK parameters will be derived in order to assess the steady state PK and the overall exposure to Rupatadine and the two main metabolites:

Plasma:

- C_{max}
- T_{max}
- AUC_{τ}, where τ = 24h post dose on Day 5
- Area under the plasma concentration vs time curve from time zero to the last quantifiable concentration (AUC_{0-t})
- $AUC_{0-\infty}$
- $t_{½}$
- Minimum plasma concentration (C_{min})
- Average plasma concentration (C_{avg})
- Mean residence time (MRT)
- Vz/F - for Rupatadine only
- CL/F - for Rupatadine only
- Accumulation ratio (Rac)

Urine:

- Ae
- CLR

Pharmacodynamic (Cognitive) Analysis

Results of cognitive tests will be listed for each subject, along with summary statistics including arithmetic and geometric means, standard deviations, minimum, maximum and median values, and coefficients of variation. Further analysis may be performed in an exploratory manner and will be documented in the SAP, as appropriate.

ECG analysis:

Heart Rate Correction: Fridericia's QT correction formula will be used to estimate the QTc interval.

Concentration Effect Relationship: Plots of the differences with 90% CIs between plasma Rupatadine, *desloratadine and hydroxylatedesloratadine* concentrations versus baseline (Day -1 data) and placebo over time will be produced for all analyses.

Assay sensitivity for the ECG analyses will be assessed by calculating the food effect in a time-course effect analysis.

Safety analysis:

AEs will be coded and reported according to MedDRA (Medical Dictionary for Regulatory Activities). All AEs will be listed by subject. The number and percentage of subjects reporting AEs, serious adverse event (SAEs), Investigational Medicinal Product (IMP)-related AEs and AEs leading to withdrawal will be summarised by treatment. Other safety parameters will also be listed by subject. Quantitative safety parameters will be summarised by treatment and time.

6. INTRODUCTION

Rupatadine is a second generation antihistamine, long-acting histamine antagonist, with selective peripheral H1-receptor antagonist activity. Some of the metabolites (desloratadine and its hydroxylated metabolites) retain an antihistaminic activity and may partially contribute to the overall efficacy of the drug. As per Summary of Product Characteristics (SmPC) for Rupatadine[1] and Investigator's Brochure[2] in vitro studies with Rupatadine at high concentration have shown an inhibition of the degranulation of mast cells induced by immunological and non-immunological stimuli as well as the release of cytokines, particularly of the TNFα in human mast cells and monocytes. The clinical relevance of the observed experimental data remains to be confirmed. Chronic idiopathic urticaria was studied as a clinical model for urticarial conditions, since the underlying pathophysiology is similar, regardless of etiology, and because chronic patients can be more easily recruited prospectively. Since histamine release is a causal factor in all urticarial diseases, Rupatadine is expected to be effective in providing symptomatic relief for other urticarial conditions, in addition to chronic idiopathic urticaria, as advised in clinical guidelines. In placebo-controlled trials in patients with Chronic Idiopathic Urticaria, Rupatadine was effective reducing the mean pruritus score from baseline over the 4 week treatment period (change vs baseline: Rupatadine 57.5%, placebo 44.9%) and decreasing the mean number of wheals (54.3% vs 39.7%).

In several Phase II and III seasonal and perennial allergic rhinitis studies, Rupatadine has demonstrated safety and efficacy. Studies carried out in 1,700 allergic rhinitis patients demonstrated that rupatadine was superior to placebo in the reduction of total symptoms. When comparing its efficacy versus other anti-H_1, rupatadine fumarate at doses of 10 and 20 mg was at least as effective as loratadine, desloratadine, ebastine and cetirizine at the doses recommended by the manufacturer.

Cardiac safety of Rupatadine was assessed in a thorough QT/QTc study. Rupatadine up to 10 times therapeutic dose did not produce any effect on the ECG and hence raises no cardiac safety concerns. Rupatadine has shown a good cardiac and CNS (central nervous system) safety profile in different clinical trials.

Rupatadine is a marketed drug indicated for symptomatic treatment of allergic rhinitis and urticaria in adults and adolescents.

6.1 Pharmacokinetic properties

6.1.1 Absorption and bioavailability

Rupatadine is rapidly absorbed after oral administration, with a T_{max} of approximately 0.75 hours after intake. The mean C_{max} was 2.6 ng/ml after a single oral dose of 10 mg and 4.6 ng/ml after a single oral dose of 20 mg. Pharmacokinetics of Rupatadine was linear for a dose between 10 and 40 mg. After a dose of 10 mg once a day for 7 days, the mean C_{max} was 3.8 ng/ml. The plasma concentration followed a bi-exponential drop-off with a mean elimination half-life of 5.9 hours. The binding-rate of Rupatadine to plasma proteins was 98.5-99%. As Rupatadine has never been administered to humans by intravenous route, no data is available on its absolute bioavailability.

6.1.2 Effect of the intake of food

Intake of food increased the systemic exposure (AUC) to Rupatadine by about 23%. The exposure to one of its active metabolites and to the main inactive metabolite was practically the same (reduction of about 5% and 3% respectively). The time taken to reach the

maximum plasma concentration (T_{max}) of Rupatadine was delayed by 1 hour. The maximum plasma concentration (Cmax) was not affected by food intake. These differences had no clinical significance.

6.1.3 Metabolism and elimination

In a study of excretion in humans (40 mg of 14C-Rupatadine), 34.6% of the radioactivity administered was recovered in urine and 60.9% in faeces collected over 7 days. Rupatadine undergoes considerable pre-systemic metabolism when administered by oral route. The amounts of unaltered active substance found in urine and faeces were insignificant. This means that Rupatadine is almost completely metabolised. In vitro metabolism studies in human liver microsomes indicate that Rupatadine is mainly metabolised by the cytochrome P450 (CYP 3A4).

6.2 Undesirable effects

Rupatadine 10 mg has been administered to over 2025 patients in clinical studies, 120 of whom received Rupatadine for at least 1 year. The most common adverse reactions in controlled clinical studies were somnolence (9.5%), headache (6.9%) and fatigue (3.2%). The majority of adverse reactions observed in clinical trials were mild to moderate in severity and usually did not require cessation of therapy. The frequencies of adverse reactions are summarised below:

**System Organ Class**	**Common (≥ 1/100 to < 1/10)**	**Uncommon (≥ 1/1000 to < 1/100)**
Nervous system disorders	Somnolence, Headache, Dizziness	Disturbance in attention
Respiratory, thoracic and mediastinal disorders		Epistaxis, Nasal dryness, Pharyngitis, Cough, Dry throat, Pharyngolaryngeal pain, Rhinitis
Gastrointestinal disorders	Dry mouth	Nausea, Abdominal pain upper, Diarrhoea, Dyspepsia, Vomiting, Abdominal pain, Constipation
Skin and subcutaneous tissue disorders		Rash
Musculoskeletal and connective tissue disorders		Back pain, Arthralgia, Myalgia
Metabolism and nutrition disorders		Increased appetite
General disorders and administration site conditions	Fatigue, Asthenia	Thirst, Malaise, Pyrexia
Psychiatric disorders		Irritability
Investigations/ tests		Blood creatine phosphokinase increased, Alanine aminotransferase increased, Aspartate aminotransferase increased, Liver function test abnormal, Weight increased

Further details on Rupatadine pharmacology, pharmacokinetics and safety can be found in the Investigator's Brochure for Rupatadine[2] and its SmPC[1].

6.3 Rationale for conducting study

Currently, Rupatadine has only been administered to non-Japanese subjects. It is therefore important to understand the safety, tolerability, PK and PD of single and repeated daily dosing of Rupatadine in Japanese subjects in order to allow development and use of the drug in Japanese patients following the current recommendation from Pharmaceuticals and Medical Devices Agency (PMDA), Japan.

6.4 Risk/benefit and ethical assessment

6.4.1 Potential Benefits

The study drug, Rupatadine, will be given to healthy subjects purely for research and development purposes and those subjects receiving study medication are not expected to benefit from the study.

6.4.2 Potential Risks

Potential risks have been identified through review of the pre-clinical and clinical studies conducted to date. Moreover, Rupatadine is a marketed drug with a very good and well established safety profile.

Risks to subjects will be minimised by applying the dose escalation procedures, study specific adaptive design features and stopping rules.

7. STUDY OBJECTIVES AND RATIONALE

7.1 Study Objectives

Primary

1. To assess the safety and tolerability of Rupatadine following single and multiple oral administrations to healthy Japanese subjects.

Secondary

1. To investigate the PK of Rupatadine and its two main metabolites desloratadine (UR 12790) and hydroxylatedesloratadine (UR 12788) in plasma and urine (including free and conjugated UR12788) following single and multiple dosing of Rupatadine once daily in healthy Japanese subjects.

2. To investigate the PD activity of Rupatadine by assessment of dose on cognitive function.

3. To measure the effect of Rupatadine on ECG parameters.

8. STUDY DESIGN

8.1 Overall study design and procedure

This will be a single centre, randomised, placebo controlled parallel group study.

The study will be conducted in three cohorts of subjects dosed with Rupatadine or matching placebo. All subjects will receive placebo on Day -1. A single dose of Rupatadine or placebo will be administered on Day 1 followed by once daily doses on Days 2 to 5.There will be no washout following the single dose on Day 1 as only PK parameters up to 24 hours will be estimated after a single dose.

Since Rupatadine is a marketed drug with a very good safety profile, there is no requirement to stagger dosing within each cohort (sentinel groups). Rupatadine was well tolerated in a clinical safety study at daily dose of 100 mg over 5 days. The starting dose of Rupatadine in this trial will be the normal therapeutic dose of 10mg. The other two anticipated dose levels are 20 mg and 30 mg. After the first and second cohort, a Safety Review Committee (SRC) will decide the dose for the next dose level, based on all cumulative data to date. For progressing to the second dose level, safety and tolerability data will be required; for progressing to the third dose level, safety tolerability and PK data will be required. If significant differences in pharmacokinetics are seen in Japanese subjects compared to the historical PK data in Caucasians, the dose for the third dose level will be decided as appropriate by the SRC. In any event, the highest dose will not exceed 100 mg Rupatadine OR a dose that is expected to yield plasma levels of Rupatadine and/or its metabolites which will not go above the levels seen in Caucasian volunteers after a 100 mg dose. The SRC may decide to hold additional SRC meetings at any time during the trial.

General eligibility of subjects for participation in this study will be assessed at screening which will take place within 21 days of the first study drug administration. Subjects will be resident from the night before the baseline assessment day (Day -2) until 96 hours after their last dose (Day 9). They will attend the unit on Days 10 and 11 (at 120 and 144 hours after last dose); their last visit will also serve as the post study follow up.

Twenty seven eligible subjects will be randomised to one of the two treatments. It is anticipated that 21 subjects will be randomised to receive single daily oral doses of Rupatadine 10 mg, 20 mg and 30 mg; (seven subjects per dose level) and that six subjects will receive matching placebo (two per dose level).

Additional subjects may be randomised to ensure that the target of obtaining Rupatadine PK data from a minimum of 6 subjects per dose level has been met. Additional subjects may be joined to a later cohort as is deemed practicable by the PI.

Assessments to be performed during the study are detailed in the study plan and assessment schedule (Tables 2 and 3).

This study will be conducted in a randomised, double-blind, placebo-controlled design with single multiple ascending doses using features of an adaptive study design. Further explanations of adaptive features which are specific to this study design are provided in Table 1, which captures all adaptive features for the entire study.

Table 1 Adaptive protocol features

Adaptive Study Design Areas	Features	Limits
**Dose**	1. All anticipated dose levels can be adjusted up or down in accordance with pharmacokinetic (PK), pharmacodynamic (PD), safety and tolerability data collected up to the decision making time-point.	1. The anticipated dose levels are 10, 20 and 30mg.<br>2. Doses are administered to dose groups in an escalating fashion.<br>3. The upper limits of the dose are defined as 100mg Rupatadine OR a dose that is expected to yield plasma levels plasma levels of Rupatadine and or its metabolites not too exceed the levels seen in Caucasian volunteers after a 100mg dose (which was well tolerated)
**Timing**	1. Straggler or replacement volunteers of a previous cohort may be run together with any of the following cohorts.	1. None
**Flexible Cohort Sizes**	1. Withdrawn subjects can be replaced at the discretion of the Sponsor and Investigator.	1. Protocol specific minimum requirements need to be met prior to dose escalation.
	2. Cohorts at selected dose levels can be extended to<br>a. gather further information on a dose level<br>b. ensure that a minimum of 6 evaluable subjects on receiving Rupatadine complete each dose level.	1. The maximum extension is 100% of the original cohort at the selected dose level when the objective is to gather further information.<br>2. When the objective of the extension is to complete a minimum of 8 evaluable subjects per dose level, the maximum extension is the number of participants to achieve that aim.

Adaptive Study Design Areas	Features	Limits
	3. Cohort size can be decreased.	1. There must be a minimum of 6 subjects completing the study for the purpose of dose escalation.<br>2. There must be a minimum of 8 subjects completing the study for the purpose of study analyses.
**Optional Cohorts**	1. None	N/A
**Samples and Assessments**	1. Additional safety blood samples may be taken if:<br>a. it is considered clinically necessary by the Investigator/Delegate for individuals on a case-by-case basis.<br>b. the SRC considers it necessary from a safety/tolerability point for an upcoming dose level/cohort if clinically indicated.	1. A maximum for individuals on a case-by-case basis cannot be defined because investigations will be performed as necessary to ensure the safety of the individual participants.<br>2. Study specific maximum blood volume taken specified in the protocol will not be exceeded.
	2. Timing of PK samples may be adjusted in accordance with evolving data.<br>3. Additional or less PK samples may be taken in accordance with evolving data and dosing schedule.	1. Minimum: Sufficient PK samples to establish full protocol specific PK profile.<br>2. Study specific maximum blood volume taken will not be exceeded.
	4. Timing of safety assessments such as vital signs and electrocardiograms (ECGs) may be adjusted in accordance with evolving data.<br>5. Additional safety and PD assessments such as vital signs, ECGs, cognitive tests may be taken in accordance with evolving data.	1. Alterations in timing of the safety assessments need to be a reflection of the established PK, safety and tolerability profile up to the decision making time-point, in particular T_{max} and $t_{1/2}$.<br>2. Alterations need to be made in the spirit of the current study protocol (i.e., focus on the capture of essential and useful data) and not affect the risk profile of the study

Adaptive Study Design Areas	Features	Limits
**Data Analysis**	1. Allow for the additional analysis of ECG Telemetry and Holter data	1. The use of the safety data set may be extended to investigate further the cardiac safety profile.

Table 2 Study Plan

	SCR	A/M	B/L	SAD	MAD								FU
**Study Day**	-21-2	-2	-1	1	2	3	4	5	6	7	8	9	10	11
Informed Consent	X													
Demographic Data	X													
Past Medical History	X	X[5]												
Physical Examination	X	X[5]												X[5]
In/Exclusion Criteria	X	X												
Weight/Height/BMI	X	X[6]												
Body Temperature	X	X												X
Vital Signs (BP/HR)[7]	X	X	X	X				X						X
Urine DoA Screen	X	X												
Alcohol Breath Test	X	X												
12-lead ECG[7]	X		X	X				X						X
Holter ECG (24 hours)	X													
12-lead telemetry ECG			X[1]	X[1]				X[1]						
Safety Blood Profile	X													X
Serology	X													
Urinalysis	X													X
Pregnancy Test (females only)	X	X												X
Drug Administration[7]			X	X	X	X	X	X						
PK Blood Sampling[7]				X	X[2]	X[2]	X[2]	X	X	X	X	X	X	X
Exploratory sample				X										
Urine Collection[7]				X	X			X	X	X	X	X		
Cognitive tests[7]		X[3]	X	X				X						
Meals[4]		X	X	X	X	X	X	X	X	X	X			
AE/ConMed Recording	X	X	X	X	X	X	X	X	X	X	X	X	X	X
Study Residence		X	X	X	X	X	X	X	X	X	X			

[1] 12-lead telemetry ECG will be performed from approximately 1 hour pre-dose until at least 6 hours post dose on Day -1, Day 1 and Day 5.

[2] Trough (pre-dose) samples only

[3] Training

[4] Meals will be served at approximately the standard Unit times as follows: breakfast (2 hours post –dose), lunch (6 hours post-dose), dinner (12 hours post-dose).

[5] confirmation and or checking for change only

[6] weight only

[7] see table 3 for assessment time points.

Table 3 Assessment Schedule

Study Day	Protocol Time (hh:mm)	Dose	PK Sampling (on time)	Urine Collection	Vital Signs[2]	ECG (PK - 5min)	12-lead Telemetry	Cog Test (after PK)	Meal	Other
**-2**	Admission							X[3]	X	X[1]
**-1**	**Pre-dose**				X	X	x-0			
	**00:00**	X[4]								
	**00:20**					X	0-6			
	**00:40**					X	0-6			
	**01:00**					X	0-6	X		
	**01:30**					X	0-6			
	**02:00**					X	0-6		X[5]	
	**03:00**					X	0-6	X		
	**04:00**					X	0-6			
	**05:00**					X	0-6			
	**06:00**					X	0-6		X	
	**08:00**					X				
	**12:00**					X			X	
**1**	**Pre-dose**		X	x-0	X	X	x-0			
	**00:00**	X								
	**00:20**		X	0-6		X	0-6			
	**00:40**		X	0-6		X	0-6			
	**01:00**		X	0-6	X	X	0-6	X		
	**01:30**		X	0-6		X	0-6			
	**02:00**		X	0-6	X	X	0-6		X[5]	
	**03:00**		X	0-6		X	0-6	X		
	**04:00**		X	0-6		X	0-6			
	**05:00**			0-6		X	0-6			
	**06:00**		X	0-6		X	0-6		X	
	**08:00**		X	6-12		X				
	**12:00**		X	6-12		X			X	
**2**	**Pre-dose**		X	12-24						
	**00:00**	X								
**3**	**Pre-dose**		X							
	**00:00**	X								
**4**	**Pre-dose**		X							
	**00:00**	X								
	**02:00**								X	
	**06:00**								X	
	**12:00**								X	
**5**	**Pre-dose**		X	x-0	X	X	x-0			
	**00:00**	X								
	**00:20**		X	0-6		X	0-6			
	**00:40**		X	0-6		X	0-6			
	**01:00**		X	0-6	X	X	0-6	X		
	**01:30**		X	0-6		X	0-6			
	**02:00**		X	0-6	X	X	0-6		X[5]	
	**03:00**		X	0-6		X	0-6	X		
	**04:00**		X	0-6		X	0-6			
	**05:00**			0-6		X	0-6			
	**06:00**		X	0-6		X	0-6		X	
	**08:00**		X	6-12		X				
	**12:00**		X	6-12		X			X	
**6**	**24:00**		X	-24						
**7**	**48:00**		X	-48						
**8**	**72:00**		X	-72						
**9**	**96:00**		X	-96						discharge
**10**	**120:00**		X							
**11**	**144:00**		X		X	X				X[1]
	**N =**	**1+5**	**31**	**11**	**8**	**37**	**3**	**7**		**2**

[1]Safety haematology and biochemistry

[2]supine blood pressure and heart rate only;

[3]Training sessions

[4]Placebo dosing only; [5]Standard carbohydrate rich meals at 2 hours post dose, standard meals 6 and 12 hours post dose, identical for days-1, 1 and 5.

8.2 Dose escalation

8.2.1 Dose escalation procedures

Mandatory formal dose escalation procedures are planned to be conducted between the first and the second dose group and between the second and third dose group. For progressing to the second dose level, safety and tolerability data will be required; for progressing to the third dose level, safety tolerability and PK data will be required. Assessment of PK data will focus on the comparison of pharmacokinetic parameters between Japanese subjects (from this study) and Caucasian subjects (historical data). Additional dose escalation procedures can be performed, e.g., in case of unexpected adverse events, to aid adjustment of doses

If PK profiles are found to be different between Japanese and Caucasian subjects (historical data) in a clinically meaningful way, the next Rupatadine dose level will be adjusted accordingly, which may be up or down, however in any case it will not exceed 100 mg OR a dose that is expected to yield plasma levels of Rupatadine and/or its metabolites which will not go above the levels seen in Caucasian volunteers after a 100 mg dose.

Progression from SAD part to MAD part within each dose group will require a clinical review of AE data following the single dose, by the Investigator or delegate. The decision will be communicated by e-mail.

8.2.2 Safety Review Committee

After each of the first two dose groups is completed, the SRC will evaluate the safety, tolerability and PK of Rupatadine and decide the next dose. There is an option to have ad-hoc SRC meetings to discuss urgent safety issues should the need arise.

The SRC will consist of:

- Sponsor’s Medical Director, Dr Iñaki Izquierdo, MD PhD, or delegate
- Sponsor’s Clinical Trial Manager, Eva Santamaria Masdeu, or delegate
- Principal Investigator (PI), Dr Jorg Taubel, MD FFPM, or delegate

Further internal or external experts may be consulted by the SRC as necessary.

8.2.2.1 Data requirements

The SRC will assess the minimum PK (for progressing to the third dose level only), safety and tolerability data required prior to dose escalation. Prior to the SRC meeting, an interim safety report will be prepared presenting the relevant safety and tolerability findings, signed by the Principal Investigator (PI) or delegate. The PK report will be provided by Richmond Pharmacology Ltd (RPL). The following safety and PK data will be required by the SRC for the formal dose escalation:

<u>Safety (</u>for progressing to the second and third dose level)
Safety data up to 96 hours after the last dose on Day 5 will be required including:

- Adverse events
- Vital signs (blood pressure, heart rate)
- 12-lead ECG
- Physical examinations

- Clinical laboratory parameters (haematology, biochemistry, urinalysis)

PK (for progressing to the third dose level only)
PK data up to 96 hours after the last dose on Day 5 (from the first and second dose level) will be required.

8.2.2.2 SRC Meeting

The SRC will determine dose escalations/progression according to the clinical study protocol, anticipated dose(s) and adaptive features. The decision for dose escalation will be signed by one of the Sponsor's representatives and one of the Investigator's representatives. For logistical reasons the decision from the Sponsor may be communicated via email.

The decision of the SRC on the next dose will be taken in consensus between the members of the SRC. If consensus cannot be reached then the most cautious approach will proceed. The decisions and decision-making of the SRC on the next dose level will be documented and provided to the PI and the RPL Pharmacy prior to the next scheduled dosing day.

All data (reports) that are provided and reviewed by the SRC including any minutes/decisions from the meeting will be archived electronically by the PI.

8.2.3 Stopping criteria

Group stopping criteria
Cohorts will escalate through three anticipated dose levels including single and multiple doses, i.e. each subject will participate in one dose level. Dose escalation procedures are described in section 8.2.1.

For the purpose of this protocol the term 'dose escalation' means escalation to the next higher dose.

For the purpose of this protocol the term 'suspension' means that further dose escalation will be suspended and that dose level will not be repeated. Any resumption at the same or higher dose levels will require a substantial amendment which has been approved by the Regulatory Authority and the Research Ethics Committee (REC).

If a dose level is well tolerated by the subjects and there are no instances of drug related clinically significant Grade II or higher adverse events reported, the next dose level(s) can be administered in the next cohort(s).

If clinically significant and drug related Grade II adverse events are observed in up to 2 study subjects in a cohort in different organ systems, the dose(s) to be administered in the next cohort(s) will be determined by the SRC and based on the nature of the adverse events observed.

If clinically significant drug related Grade II adverse events are observed in 2 subjects in the same organ system or 3 subjects in different organ systems, the dose will in the first instance not be further escalated. Dosing at the relevant dose level may continue and the dose level's cohort may be extended in accordance with Table 1 to gather additional information prior to further dose escalation. The dose may be escalated if, after continuation of the dose level and or extension of the cohort, the minimum dose escalation requirements are met and the stopping criteria in the next paragraph are not met.

If clinically significant drug related Grade II adverse events are observed in 3 or more trial subjects in the same organ system or 4 or more subjects in different organ systems, dosing in the given cohort will be suspended and the dose will not be further escalated.

A lower (intermediate) dose level may be administered in the next cohort at the discretion of the SRC.

Exceptions

Grade II adverse events will be considered as clinically significant drug-related **unless:**

1. The adverse events are clearly unrelated to the treatment, Investigational Medicinal Product (IMP) or trial procedures
2. The adverse events are expected drug effects due to the mode of action of the compound and do not represent a greater than the anticipated minimal safety risk to the subject(s). Expected drug effects are described in the IB.
3. Adverse events are commonly observed and therefore expected adverse events in healthy subject trials where a relationship to the trial treatment or medication is unlikely but cannot be fully excluded and which do not present a significant clinical risk to the subjects such as pre-syncope, vasovagal reaction, anxiety, headache and nausea.

In cases where the above exceptions apply, dose escalation or extension of a dose level's cohort can proceed if the SRC agrees to do so.

If clinically significant drug-related Grade III adverse events which are not classified as serious are observed in 2 or more subjects in the same organ system or 3 or more subjects in different organ systems, dosing in the given cohort will be suspended and the dose will not be extended or further escalated.

In such case, a lower (intermediate) dose level may be administered in the next cohort at the discretion of the SRC.

If any clinically significant drug-related Grade III adverse events classified as serious or any drug related Grade IV (life-threatening) or V (fatal) adverse events occur, dosing in the given cohort will be suspended and the dose will not be extended or further escalated.

In case of clinically significant drug-related Grade III adverse events classified as serious (but not clinically significant drug-related Grade IV or V serious adverse events) a lower (intermediate) dose level may be administered in the next cohort, if considered appropriate by the SRC after consultation with relevant external experts.

Table 4 Group stopping criteria based on clinically significant drug-related adverse events

Group Stopping Criteria				
**Grade**	**Severity/Seriousness**	**Number of Subjects affected**	**Action**	**Effect on dose progression or escalation**
I	Mild		No action required.	
II*	Moderate	≤2 subjects in different SOC.**	Next dose determined by SRC.	
		≤2 subjects in same SOC.<br>3 subjects in different SOC.	Dose level may continue.<br>Dose level may be extended.<br>Dose escalation on hold until results of continuation and or extension available.	Following continuation and/or extension dose escalation may proceed as per protocol, unless stopping criteria below are met.
		≥3 subjects in same SOC.<br>≥4 subjects in different SOC.	Dosing suspended.<br>Dose escalation or extension prohibited.	A lower (intermediate) dose level may be administered in the next cohort.<br><br>Dose continuation, extension or escalation requires substantial amendment.
III	Severe, not serious	≤2 subjects in different SOC.	Next dose determined by SRC.	
		≥2 subjects in same SOC.<br>≥3 subjects in different SOC.	Dosing suspended.<br>Dose escalation or extension prohibited.	A lower (intermediate) dose level may be administered in the next cohort.<br><br>Dose continuation, extension or escalation requires substantial amendment.
	Severe, serious	≥1 subject	Dosing suspended.<br>Dose escalation or	A lower (intermediate) dose level may be

			extension prohibited.	administered in the next cohort.<br><br>Dose continuation, extension or escalation requires substantial amendment.
IV	Life-threatening	≥1 subject	Study suspended	Study continuation requires substantial amendment.
V	Fatal	≥1 subject	Study suspended	Study continuation requires substantial amendment.

* Exceptions apply for Grade II adverse events , please refer to section 8.2.3

** System Organ Class(es)

Individual stopping criteria

Dose administration will be discontinued in a subject if any of the following events occur in any cohort:

1. Any subject experiencing clinically significant drug related Grade II adverse events will be discontinued from the trial unless meeting the above exception criteria for Grade II adverse events (in which case the subject can continue at the Investigator's discretion).
2. Any occurrence of clinically significant drug related Grade III adverse event or a drug related serious adverse event (SAE) or suspected unexpected serious adverse reaction (SUSAR) will suspend further dosing in that subject and the subject should be discontinued.

Table 5 Individual stopping criteria based on clinically significant drug related adverse events

**Individual stopping criteria**		
**Grade**	**Severity/Seriousness**	**Action**
I	Mild	No action required
II*	Moderate	Subject will be discontinued from the study unless exceptions apply, in which case subject can continue at Investigator's discretion
III	Severe, not serious	Subject will be discontinued from the study
	Severe, serious	Subject will be discontinued from the study
IV	Life-threatening	Subject will be discontinued from the study
V	Fatal	

*Exceptions apply for Grade II adverse events, please refer to section 8.2.3.

Standard toxicity grading according to the National Cancer Institute Common Terminology Criteria for Adverse Events will be used to grade the adverse events (NCI CTCAE version 4.0). Local laboratory normal values will be applied. Abnormal laboratory and other tests will be repeated prior to grading in order to ensure consistency and to exclude technical errors. Diurnal variations in laboratory parameters and other measurements as well as baseline status and conditions (e.g. Gilbert's syndrome) will be taken into account when assessing whether abnormalities constitute a drug related Adverse Event and when grading, if applicable.

The CTCAE displays adverse event/toxicity grades I through V with detailed clinical descriptions of severity for individual adverse events based on a general guideline. The general grade definitions are described as follows:

Grade 1: Mild; asymptomatic or mild symptoms; clinical or diagnostic observations only; intervention not indicated

Grade 2: Moderate; minimal, local or non-invasive intervention indicated; limiting age-appropriate instrumental ADL*

Grade 3: Severe or medically significant but not immediately life-threatening; hospitalization or prolongation of hospitalization indicated; disabling; limiting self care ADL**

Grade 4: Life-threatening consequences; urgent intervention indicated

Grade 5: Death related to AE

*Instrumental Activities of Daily Living (ADL) refer to preparing meals, shopping for groceries or clothes, using the telephone, managing money, etc.
**Self care ADL refer to bathing, dressing and undressing, feeding self, using the toilet, taking medications, and not bedridden.

The CTCAE criteria and their interpretation are consistent with the standard intensity grading for adverse events during clinical trials: Grade I: mild, Grade II: moderate, Grade III: severe or medically significant but not immediately life-threatening, may constitute SAE/SUSAR. Grades IV and V constitute SAE/SUSAR.

The grading of the NCI CTCAE criteria related to target organ toxicities and potentially expected adverse events (including study related sections on: 'hepatobiliary disorders', 'investigations' - specifically liver function tests - , 'gastrointestinal disorders', such as 'abdominal distension', 'abdominal pain', 'diarrhoea', 'nausea' is considered suitable for this particular IMP, study design and the study populations in conjunction with the protocol stopping rules and no further qualifications are required.

8.3 Rationale for study design, doses and control groups

A single and multiple ascending dose design is used for this study to evaluate the safety, tolerability, PD and PK of the investigational product in healthy subjects. The study is randomised and double blind to minimise bias and includes placebo to facilitate distinguishing effects related to administration of drug from those related to the study procedures or situation. No wash out between SAD (Day 1) and MAD part (Days 1-5) was built into the study design since 24 hours is deemed sufficient to characterise the Rupatadine

pharmacokinetics. Twenty four hours after the first dose may not be sufficient for characterisation of the Rupatadine metabolites PK. However, steady state pharmacokinetics are considered more relevant for the metabolites and will be comprehensively assessed after the last dose on Day 5.

8.3.1 Choice of subjects for study

The study will be conducted in healthy Japanese subjects. The selection criteria are defined such that subjects selected for participation in the study are known to be free from any significant illness to avoid interference from disease processes or other drugs.

Rupatadine has already been administered to non-Japanese subjects. In this study, it will be administered to healthy Japanese subjects in order to understand the safety, tolerability, PK and PD of single and multiple ascending doses in this population in order to allow development and use of the drug in Japanese patients.

8.3.2 Route and rate of administration

Rupatadine will be administered via the oral route as this will be the route of administration for patients.

To date, Rupatadine has been administered in single and multiple oral dose studies with doses ranging from 2 mg to 100 mg. The anticipated doses for this study are well within this range and are deemed safe to be administered to healthy Japanese and Caucasian subjects.

Dose escalation will proceed in accordance with Table 1 of adaptive features and will not exceed 100 mg.

8.3.3 Estimation of first dose in human subjects

Not applicable, as this is not First into Humans study.

8.3.4 Precautions to be applied for dosing between subjects within a cohort

Not applicable for this study.

8.3.5 Precautions to be applied for dosing between different cohorts

See Section 8.2.

8.3.6 Dose escalation scheme

See Section 8.2.

8.3.7 Stopping criteria

See Section 8.2.

8.3.8 Monitoring and communication of adverse events / reactions

AEs will be continuously monitored throughout the study from first dose until the last follow up assessment. Each AE reported will be assessed by a trained Research Physician who will ensure that the event is dealt with as appropriate based on clinical need, study protocol and the clinical pharmacology unit (CPU) standard operating procedures (SOPs). Adverse

events will be documented in the subjects' Case Report Forms (CRFs) and reviewed regularly by the Research Physicians and the Investigator.

If any information relating to the study drug in this study becomes available after the submission of a final protocol to the Competent Authority which may impact on the conduct of the study, including but not limited to the risk and benefit evaluations underpinning approvals and volunteer's consent, J. Uriach y Compañía, S.A. shall notify RPL in writing as soon as practically possible and the parties will agree, in writing, what steps need to be taken, if any.

8.3.9 Investigator site facilities and personnel

This study will be conducted in a specialised early phase CPU within an acute hospital setting with Critical Care facilities, thus ensuring direct access to equipment and staff for resuscitating and stabilising subjects in acute medical conditions and emergencies. The study is conducted by an experienced PI and well trained medical, nursing and technical staff with ample experience in the conduct of early phase clinical trials.

The study is designed to closely monitor, treat and communicate potential expected adverse reactions (based on the known mode of action of the IMP and the previous studies with similar compounds) as well as potential unexpected adverse events.

9. SELECTION AND WITHDRAWAL OF SUBJECTS

9.1 Number and Source of Subjects

For the three planned dose levels, it is intended that a total of 27 complete evaluable healthy Japanese subjects will be randomised.

Volunteers to participate in the study will be recruited from RPL's volunteer database and general population using web advertising and other media as appropriate.

Volunteer screening will include tests stipulated in the study plan (Table 2). If results for any of the required tests are available from other source, they can be used for screening provided they are in accordance with the protocol.

9.2 Inclusion Criteria

Subjects will be eligible to participate in this clinical study if they meet the following criteria at the screening and/or admission visit:

1. Healthy male or female Japanese subject, aged 20 - 45 years, inclusive, at screening.

   Japanese subjects must meet the following criteria: i) born in Japan to both Japanese parents and grandparents; ii) lived less than 5 years outside of Japan; iii) no significant change in lifestyle, including diet, since leaving Japan

2. Subject has a Body Mass Index (BMI) of 18 – 25 kg/m^2 inclusive at screening.

3. Subjects must agree to use acceptable methods of contraception:

   If female, subjects of childbearing potential must agree to use medically acceptable methods of contraception from the time of signing the informed consent until

3 months following administration of the last treatment or dose of study medication as outlined below:

- A documented placement of an intrauterine device (IUD) or intrauterine system (IUS) and the use of a barrier method {condom or occlusive cap (diaphragm or cervical/vault caps) used with spermicidal foam/gel/film/cream/suppository};
- Oral contraceptives (combination oestrogen/progesterone pills), injectable progesterone or subdermal implants and the use of a barrier method {condom or occlusive cap (diaphragm or cervical/vault caps) used with spermicidal foam/gel/film/cream/suppository};
- Documented tubal ligation (female sterilization). In addition, a barrier method [condom or occlusive cap (diaphragm or cervical/vault caps) used with spermicidial foam/gel/film/cream/suppository] should also be used;
- Double barrier method: Condom and occlusive cap (diaphragm or cervical/vault caps) with spermicidal foam/gel/film/cream/suppository;
- True abstinence: When this is in line with the preferred and usual lifestyle of the subject. [Periodic abstinence (e.g., calendar, ovulation, symptothermal, post-ovulation methods) and withdrawal are not acceptable methods of contraception].

Male subjects must utilise at least one of the following:

- use a condom with spermicidal foam/gel/film/cream/suppository if their female partner(s) is (are) pregnant or lactating from the time of the first administration of treatment or study medication until 3 months following administration of the last treatment or dose of study medication.
- Use acceptable methods of contraception if the male subject's partner could become pregnant from the time of the first administration of treatment or study medication until 3 months following administration of the last treatment or dose of study medication. The acceptable methods of contraception are as follows:
- Condom and occlusive cap (diaphragm or cervical/vault caps) with spermicidal foam/gel/film/cream/suppository;
- Surgical sterilisation (vasectomy with documentation of azoospermia) and a barrier method {condom or occlusive cap (diaphragm or cervical/vault caps) used with spermicidal foam/gel/film/cream/suppository};
- The female partner uses oral contraceptives (combination oestrogen/progesterone pills), injectable progesterone or subdermal implants and a barrier method {condom or occlusive cap (diaphragm or cervical/vault caps) used with spermicidal foam/gel/film/cream/suppository};
- The female partner uses medically prescribed topically-applied transdermal contraceptive patch and a barrier method {condom or occlusive cap (diaphragm or cervical/vault caps) used with spermicidal foam/gel/film/cream/suppository};
- The female partner has undergone documented tubal ligation (female sterilisation). In addition, a barrier method {condom or occlusive cap (diaphragm or cervical/vault caps) used with spermicidal foam/gel/film/cream/suppository} must be used;
- The female partner has undergone documented placement of an IUD or IUS and the use of a barrier method {condom or occlusive cap (diaphragm or cervical/vault caps) used with spermicidal foam/gel/film/cream/suppository};
- True abstinence: When this is in line with the preferred and usual lifestyle of the subject. [Periodic abstinence (e.g., calendar, ovulation, symptothermal, post-ovulation methods) and withdrawal are not acceptable methods of contraception].

Subjects should not donate egg and sperm from the time of the first administration of treatment or study medication until 3 months following administration of the last treatment or dose of study medication.

4. All subjects included in the study must meet the ECG screening selection criteria. ECG criteria to be signed off for inclusion by a cardiologist (if applicable).

5. Subjects must be capable of understanding and complying with the requirements of the protocol and must have signed the informed consent form prior to undergoing any study-related procedures.

9.3 Exclusion Criteria

Subjects will be prohibited from participation in this clinical study if they meet any of the following criteria at the screening and/or admission visits:

1. Subject has a clinically significant disease or any condition or disease that might affect drug absorption, distribution or excretion.
2. Any clinically significant abnormal laboratory, vital signs or other safety findings as determined by medical history, physical examination or other evaluations conducted at screening or on admission.
3. Electrocardiogram (ECG) abnormalities in the standard 12-lead ECG (at screening) which in the opinion of the Investigator is clinically relevant or will interfere with the ECG analysis.
4. History or current evidence of any clinically relevant cardiovascular, pulmonary, hepatic, renal, gastrointestinal, haematological, endocrinological, metabolic, neurological, psychiatric or other disease.
5. Positive results in any of the serology tests for Hepatitis B Surface Antigen (HbsAg), anti-Hepatitis core antibody (anti-HBc Ig G [and anti-HBc IgM if IgG is positive], Hepatitis C antibodies (anti-HCV), and Human Immunodeficiency Virus (HIV) 1 and 2 antibodies, (anti-HIV 1/2).
6. Confirmed positive results from urine drug screen (amphetamines, benzodiazepines, cocaine, cannabinoids, opiates, barbiturates, and methadone) or from the alcohol breath test at screening and on admission (Day -1).
7. History or clinical evidence of alcohol or drug abuse. Alcohol abuse is defined as regular weekly intake of more than 21 units (Using alcohol tracker http://www.nhs.uk/Tools/Pages/NHSAlcoholtracker.aspx); drug abuse is defined as compulsive, repetitive and/or chronic use of drugs or other substances with or without problems related to their use and/or where stopping or a reduction in dose will lead to withdrawal symptoms.
8. Mentally handicapped.
9. Participation in a drug trial within 90 days prior to first drug administration.
10. Use of any medication (including over-the-counter (OTC) medication) within 2 weeks prior to admission (Day -1) or within less than 10 times the elimination half-life of the respective drug, or anticipated concomitant medication during the treatment periods. Single intake of a drug may be accepted if judged by the investigators to have no clinical relevance and no relevance for the trial objectives.

11. Use of any substance inhibiting CYP3A4 enzymes within 2 weeks prior to admission (Day -2).
12. Donation of more than 500 mL of blood within 90 days prior to drug administration.
13. Subjects who smoke more than 10 cigarettes or equivalent amount of tobacco per day and/or who cannot stop smoking for the duration of the study whilst in the CPU.
14. Treatment with herbal supplements during the 7 days prior to dosing, or use of vitamins during 48 hours prior to admission to the CPU (Day -2).
15. Any circumstances or conditions, which, in the opinion of the PI, may affect full participation in the trial or compliance with the protocol.
16. Legal incapacity or limited legal capacity at screening.
17. Subjects who are vegetarians, vegans or have any dietary restrictions conflicting with the study standardised menus.

9.4 Subject Restrictions

Subjects will have to comply with the following restrictions during the study:

Whenever subjects are confined in the ward, only the drinks and meals provided by the trial personnel will be allowed.

Subjects will have to fast for at least 8 hours prior to the study drug administration on Day -1 and on Days 1-5. On dosing days, standardised meals will be served at the following times: breakfast (2 hours post –dose), lunch (6 hours post-dose), dinner (12 hours post-dose) as stated in the assessment schedule (Table 3). On other in-house study days, standardised meals will be provided at approximately the standard Unit times.

On Day -1, Day 1 and Day 5, water is not permitted from one hour pre-dose to four hours post-dose, save for the volume given with the study drug and liquids given with the scheduled meals. At other times water is permitted ad libitum.

Subjects must remain on bed rest in semi recumbent position for six hours post-dose on Day -1, Day 1 and Day 5, except for the 10 minute period preceding their ECG assessments and during cognitive testing.

Intake of alcohol will not be allowed 72 hours before screening and then from 72 hours prior to admission to the CPU until after final assessments on Day 11.

As per selection criteria all subjects must be non-smokers or smoke less than 10 cigarettes a day. Smoking is not allowed during the in-house period.

Intake of caffeine will not be allowed 48 hours before screening and then from 48 hours prior to admission to the CPU on Day -2 until after the final assessments on Day 11.

As per selection criteria, use of any substance inhibiting CYP3A4 enzymes will not be allowed from 2 weeks prior to admission (Day -2) until after the final assessments on Day 11.

Blood donation will not be allowed at any time during the study and up to three months (90 days) after completion of the study.

The subjects must refrain from strenuous physical exercise from 48 hours before the screening visit until after the final assessments on Day 11.

Subjects must not consume poppy seeds 48 hours before screening and then from 48 hours prior to admission to the CPU until after the final assessments on Day 11.

The subjects will have to refrain from apple juice, grapefruit, grapefruit juice, and Seville oranges from 7 days prior to admission to the CPU until after the final assessments on Day 11.

The subjects must also abstain from the consumption of energy drinks containing taurine or glucuronolactone 48 hours before screening and then from 48 hours prior to admission to the CPU until after the final assessments on Day 11.

For the restrictions related to concomitant medication, please refer to the Section 10.8 below.

9.5 Withdrawal of Subjects

9.5.1 Criteria for withdrawal

In accordance with the Declaration of Helsinki, subjects will be free to withdraw from the study at any time if they wish so, for any reason specified or unspecified.

The following reasons will be accepted for study discontinuance on by the Investigator:

– withdrawal of subject consent, or loss to follow-up, or inability to remain under medical observation including post study examination;

– severe non-compliance or major deviation from the protocol;

- any other situation where, in the opinion of the Investigator, continuation of the study would not be in the interest of the subject;

Should any of the subjects be withdrawn from the study, the Sponsor's representative and Investigator will discuss the possibility of replacement. All SAEs judged by either the Investigator or J. Uriach y Compañía, S.A. as having a reasonable suspected causal relationship to an IMP (i.e., definitively, probably or possibly related) will be qualified as serious adverse reactions. The reason for withdrawal has to be recorded in the CRF for all withdrawn subjects.

The Case Report Form (CRF) has to be completed up to the time of drop out. All drop outs after the first intake of investigational product should be given a post-study assessment as appropriate. The premature termination form in the CRF must be completed for all dropouts. Subjects withdrawn because of adverse experiences will undergo a physical examination, laboratory tests and all discharge procedures planned at discharge. In addition, a blood sample for PK analysis will be taken for every premature withdrawal. A follow-up of AEs will also be undertaken for withdrawn subjects.

Replacement subjects may be included and these subjects may be randomised to meet the minimum data requirements for the SRC.

9.5.2 Procedures for subject withdrawal

Subjects who discontinue should always be asked about the reason(s) for their discontinuation and the presence of any adverse events. If possible, they should be seen

and assessed by an Investigator(s). Adverse events should be followed up and duly documented.

If a subject is being withdrawn due to a suspected infection in World Health Organisation (WHO) risk categories 2, 3, and 4, no biological samples from this subject are allowed to be sent to the laboratory. Samples will be destroyed according to normal routines at the study site.

10. STUDY AND CONCOMITANT TREATMENTS

10.1 Investigational Medicinal Products (IMPs)

The following IMPs will be supplied to the clinical pharmacology unit for use on the study:

- Rupatadine 10 mg tablets for oral administration
- Placebo to match Rupatadine 10 mg tablets for oral administration

The Sponsor will ensure that the investigational product is manufactured in compliance with Good Manufacturing Practice (Annex XIII) and all relevant regulations. The Investigator must be familiar with the appropriate use of the study medication and ensure that it is used only in accordance with the protocol.

10.2 Doses and treatment regimens

Anticipated treatments/ dose levels are given below:

Treatment/ Dose Level	IMP dispensed	Administration
Rupatadine 10 mg	1 x Rupatadine 10 mg tablet	IMPs will be administered orally with approximately 240 mL of filtered tap water at room temperature. The subject will be in the sitting position during the administration.
Rupatadine 20 mg	2 x Rupatadine 10 mg tablet	
Rupatadine 30 mg	3 x Rupatadine 10 mg tablet	
Placebo	Number of placebo tablets to match number of Rupatadine tablets at each dose level	

On Day -1, each subject will receive a single dose of placebo. On Days 1 to 5, a total of 5 doses of Rupatadine or placebo will be administered to each subject according to the randomisation schedule (one single daily dose on Day 1, followed by single daily doses on Days 2-5). Oral doses will be administered by a Research Physician and the details of dosing will be recorded in the CRF. The dosing will be verified by another member of the Investigator's staff.

10.3 Packaging and Labelling of IMPs

The Sponsor will supply Rupatadine and placebo tablets in bulk to the Clinical Pharmacology Unit. The Unit's clinical trials pharmacy will package and label the individual subject doses.

The packaging and labelling operations will be carried out in accordance with Good Manufacturing Practice (GMP) and Annex XIII of European Union (EU) GMP Guidelines.

10.4 Drug Accountability

A medication supply form will be provided to the clinical pharmacology unit and signed by the personnel responsible for drug storage and accountability at the trial site. All drug supplies for the clinical trial must be kept in a safe and adequate place during the clinical trial.

All medication supplies (empty containers as well as partly used and unused medication) must be available for checking to the Sponsor's Monitor during the monitoring visit(s).

At the end of the study, all medication supplies (used and unused) will be reconciled at RPL's pharmacy and stored until destruction or return to the Sponsor, neither of which can take place without prior check of drug accountability by the Sponsor's Monitor and its authorisation.

10.5 Randomisation

Randomisation codes will be generated using SAS™ PROC Plan by an RPL statistician in accordance with the protocol and RPL SOPs.

10.6 Allocation of Subject Numbers

Each volunteer who signs an ICF for this study will receive an RPL Screening number generated in the volunteer database.

Eligible subjects will be randomised to receive study medication on Day -1 and assigned Subject Numbers as follows:

- Cohort 1: 101 onwards
- Cohort 2: 201 onwards
- Cohort 3: 301 onwards

Any replacement subjects will receive the subject number from the subject they are replacing + 1000 (e.g. replacement for subject number 101 would be subject number 1101).

For further details please refer to the SOM.

10.7 Blinding and Procedures for Unblinding the Study

10.7.1 Methods for ensuring blinding

This study is double-blind with regard to treatment (Rupatadine or placebo) at each dose level. During the study, the randomisation list will be kept in a locked cabinet in RPL's Pharmacy with access to authorised site personnel only including the pharmacy personnel preparing the IMPs and Qualified Person (QP). A copy of the randomisation list will also be provided to the analytical laboratory for analysis of PK samples. Upon completion of the study, once the database is locked and the blind revealed, the randomisation list is to be filed electronically and/or in the Master Study File (MSF) and archived by RPL.

10.7.2 Methods for unblinding the study

Envelopes with individual treatment codes, indicating the treatment randomisation for each randomised subject, will be available to the Investigators at the study centre and to the Sponsor.

The individual treatment code should not be broken except in medical emergencies or if the SRC decides to unblind when the appropriate management of the subject requires knowledge of the treatment randomisation. The Investigator must document and report to the Sponsor any breaking of the treatment code.

The Sponsor retains the right to break the code for SAEs that are unexpected and are suspected to be causally related to an investigational product and that potentially require expedited reporting to regulatory authorities. Treatment codes will not be broken for the planned analyses of data until all decisions on the evaluability of the data from each individual subject have been made and documented.

10.8 Concomitant Medications

Except where mentioned, no prescription or non-prescription drugs are permitted while the subjects participate in the study, except when necessary to treat an AE or in case of rescue medication.

Oral paracetamol (not exceeding 1 g per day) for the treatment of AEs will be permitted during the study.

Before using any concomitant medication, the Investigator or his representative will be consulted.

Details of previous and concomitant treatments should be recorded throughout the study by the PI or delegate on the appropriate pages of the CRF.

11. STUDY PROCEDURES

11.1 Schedule of Study Procedures

The study procedures are described in the sections below and the timing of these procedures are detailed in the Study Plan and the Assessment Schedule (Table 2 and Table 3, respectively). It is important that PK sampling occurs as close as possible to scheduled time. The sequence of assessments and allowable time windows at particular time-points will be described in the SOM.

12. STUDY METHODOLGY

12.1 Meals

Standardised meals will be provided on Day -1, Day 1 and Day 5 at the times stated in the assessment schedule (Table 3); breakfast provided at 2 hours post-dose on these days will be a carbohydrate rich meal. On other in-house study days, standardised meals will be

provided at regular CPU meal times. The details of standardised meals including their caloric and nutritional content will be provided in SOM.

12.2 Vital Signs

Blood pressure and heart rate will be measured in supine position after the subject has rested comfortably for at least 5 minutes, using automated Criticon Dynamap® monitors. Vital signs (blood pressure, and heart rate) will be measured at the time points as detailed in the assessment schedule (Table 3). Temperature will measured using tympanic thermometers at the time-points stated in the assessment schedule (Table 3).

12.3 Standard 12-lead ECG Recordings

12-lead ECGs will be recorded during the time-points described in the assessment schedule (Table 3) using a GE Marquette MAC1200® /MAC1200ST® recorder connected via a fixed network connection to the MUSE® Cardiology Information System (MUSE). ECGs recorded will be stored electronically on the MUSE information system. Only ECGs recorded electronically will be valid ECGs for any purpose other than safety assessment. ECG printouts may be filed in the subject's CRF for medical safety reviews.

Each ECG recorder will be set up to the required technical specifications and containing the information required to identify the records. Each ECG recording will be clearly identified (Subject ID, visit date, and the actual times of ECG recordings).

12-lead ECG recordings will be made at each specified time point after the subjects have been resting in a supine position for at least 10 minutes. The subjects will avoid postural changes during the ECG recordings and clinical staff will ensure that subjects are awake during the ECG recording. At each time point, triplicate 12-lead ECGs will be recorded.

All recorded ECGs will be reviewed by a Research Physician and the review will be documented in the CRF. If a subject shows an abnormal ECG, additional safety recordings (including the use of 5 or 12 lead Holter equipment) may be made and the abnormality followed to resolution if required.

12.3.1 Cardiologist over-reading of standard 12-lead ECGs

Each electronic ECG will contain the ECG data as well as the result of the automated ECG analysis performed by the Marquette® 12SL™ ECG Analysis Program (MEAP), a programme resident in each of the ECG machines.

All ECG and their associated automated interval measurements will subsequently be reviewed by qualified Cardiologists in accordance with the ICH E14 Guidance for Industry document and ICH E14 Implementation Working Group Questions and Answers document before any of the ECG are used for the thorough ECG analysis. The manual adjudication process applied in this study is also referred to in the ICH guidance and relevant literature as “manual over-read”, “computer-assisted” or “semi-automated” ECG measurements.

The following parameters on each ECG will be assessed by a cardiologist using the commercially available MUSE® in its latest version:

- QT interval
- RR interval/ Heart rate (HR)
- PR interval
- Presence or absence of U-wave

- Quantitative and qualitative ECG variations

Manual on-screen over-reading using electronic callipers in MUSE® will be performed by a small and select group of cardiologists with extensive experience with manual QT measurement (including on-screen measurement with electronic callipers). For all study ECGs, the over-reading cardiologists will be blinded to time, date, treatment and any data identifying the subject. All ECG of a given subject will be over-read by the same cardiologist (or cardiologists in case manual adjustments of the automated measurement are necessary).

12.4 Telemetry ECG Recordings

A 12-lead real time ECG will be displayed from approximately 1 hour pre-dose until 6 hours after dosing as described in the study plan (Table 2), using a Surveyor™ 12-lead system (Mortara Instrument Inc.). ECG telemetry will be monitored by the research physician, research nurse or delegate. The system will be managed according to local working practices. This assessment is used only for safety monitoring, only start and stop time is recorded in the database, and no data will be recorded in the clinical study database. The electronic ECG telemetry files will be archived and remain available for review should it be required at a later date.

12.5 Holter ECG Recordings

Holter recording will be performed at screening as described in the study plan (Table 2) using a Zymed DigiTrack Plus® Digital Holter Recorder, a compact Holter ambulatory ECG device that records and stores continuous ECGs. After the recording is finished and the device detached from the subject, the data will be downloaded and analysed using Philips 2010 Plus software. The Holter reports will be reviewed and signed off by a qualified cardiologist. In case they are available at screening, Holter reports will be valid if no older than 3 months. The screening Holter recordings will not be entered into the data base.

12.6 Physical Examination, Height and Weight

The physical examination performed at screening will include an assessment of the following: general appearance, skin, eyes, ears, nose, neck, lymph nodes, throat, heart, lungs, abdomen, musculo-skeletal system and extremities.

The physical examination performed on admission (Day -2) and on Day 11 will be a brief examination focusing on any changes since screening.

Height will be measured in centimetres and weight in kilograms. Measurements should be taken with subjects wearing light clothing and without shoes using calibrated scales for all measurements. BMI will be calculated from the height and weight.

12.7 Safety Laboratory Tests

12.7.1 Haematology and biochemistry

Blood samples for determination of haematology and biochemistry parameters will be taken at the times given in the study flowchart (Table 2). The date and time of collection will be recorded on the appropriate CRF pages. The analyses will be done at The Doctors Laboratory (TDL), using routine methods.

Blood samples for determination of standard haematology parameters will be collected in 4 mL EDTA tubes and blood samples for determination of standard biochemistry parameters will be collected in 5 mL SST tubes.

Laboratory values outside the reference limits, which are suspected to be of any clinical significance, will be repeated. Subjects in whom the suspected clinical significance is confirmed on repeated sampling will either not be included or, if already included, may be withdrawn from further participation in the study in accordance with the stopping rules in section 8.2.3 and/or followed until normalisation or for as long as the Investigator considers necessary.

Laboratory parameters to be measured are presented in Table 6.

12.7.2 Serology

Serology will be performed at Screening as detailed in the study plan (Table 2). At the screening visit all subjects will be tested for the parameters listed in Table 6. This is done for the safety of the study personnel and the result from the tests will not be entered into the study database. If a volunteer is found to be confirmed positive in any of these tests, he/she will be referred for further examination/treatment and will not be included in the study. The serology tests will be analysed from the same blood sample used for biochemistry (collected in a 5 ml SST tube). The samples will be analysed by TDL.

12.7.3 Urinalysis

Urine samples for determination of urinalysis parameters will be taken at the times given in the study plan (Table 2) and analysed at RPL. If deemed necessary, microscopic examination of urine will be performed by TDL.

12.7.4 Pregnancy test

To exclude pregnancy, a urine pregnancy test will be performed as stated in Table 2 and whenever pregnancy is suspected. Any subject with a positive pregnancy test will be excluded or withdrawn.

12.7.5 Drugs of Abuse

Urine will be tested for the drugs of abuse at RPL as described in the study plan (Table 2). If a subject fails the drugs of abuse screen, they will be excluded from the study. A repeat drug screen can only be done where methodological reasons are believed to have led to a false positive. Borderline positive results, unless covered by the preceding condition, are to be considered as positive and the subject will be excluded from the study. If subjects are found to be positive due to medication e.g. flu/cold remedies, they may undergo a repeat drug screen if they are still within the screening window. The results from the tests will not be entered into the database.

Table 6 Safety Laboratory Parameters

**Biochemistry**	**Haematology**
Aspartate aminotransferase	Red blood cells
Alanine aminotransferase	Haemoglobin
Alkaline phosphatase	Haematocrit
Lactate dehydrogenase	Mean corpuscular volume
Creatine kinase	Mean corpuscular heamaglobin concentration
Gamma GT	White blood cells
Total bilirubin	Neutrophils
Creatinine	Lymphocytes
Urea	Monocytes
Total Protein	Eosinophils
Albumin	Basophils
Glucose	Platelet count
Sodium	
Potassium	**Urinalysis***
Calcium	Leukocytes
Phosphate	Nitrite
Cholesterol	Urobilinogen
Triglycerides	Protein
	pH
	Blood
**Serology (only at screening)**	Specific gravity
Hepatitis B surface antigen	Ketones
Hepatitis C antibodies	Bilirubin
HIV 1 and 2 antibodies	Glucose
	**Urine Drugs of Abuse test**
	Amphetamines
	Barbiturates
	Benzodiazepines
	Cannabinoids
	Opiates
	Cocaine
	Methadone

* In case of out of range findings in the urinalysis an investigation of the urine microscopy may be performed.

12.8 Alcohol Breath Test

An alcohol breath test will be done at RPL using an alcometer (for time-points see the study plan [Table 2]). The results from this test will not be entered into the clinical study database. Subjects testing positive will be excluded from the study.

12.9 Cognitive Testing

Subjects will be asked to undergo battery of tests at the time points detailed in Table 3. These tests will include the following:

- Rapid Visual Information Processing (RVP)

- Reaction Time (RTI)
- Spatial Working Memory (SWM)
- Visual Analogue Scales (VAS)

All subjects will attend two training sessions on Day -2, following which the tests will be performed as per the assessment schedule (Table 3). Further details regarding the individual tests will be described in the SOM.

12.10 Pharmacokinetic (PK) Assessments

For timing of individual PK samples refer to the assessment time schedule (Table 3). The date and time of blood sample collection will be recorded in the CRF.

12.10.1 Collection of PK Samples

Venous blood samples for the determination of concentrations of Rupatadine and its two main metabolites desloratadine (UR 12790) and hydroxylatedesloratadine (UR 12788) in plasma will be taken in lithium heparin tubes at the times presented in the assessment time schedule (Table 3). For blood volume see section 12.12. Samples will be collected, stored and shipped as detailed in a separate SOM.

12.10.2 Urine Collection

Urine samples (Two aliquots of approximately 3 mL each i.e. 6 mL in total, in polypropylene tubes) for determination of concentration of Rupatadine and its two main metabolites UR 12790 and UR 12788 in urine will be taken from the total urine sample provided during each collection period presented in the study plan (Table 2). With the exception of the 0 hour (pre-dose) sample, total volume assessed by weight (g) of each urine collection interval will be recorded. Samples will be collected, stored and shipped as detailed in a separate SOM.

12.10.3 Determination of Drug Concentrations in PK samples

Plasma and urine samples for determination of the concentration of Rupatadine and its two main metabolites UR 12790 and UR 12788 will be analysed by Laboratorios Echevarne on behalf of J. Uriach y Compañía, S.A. using a validated method which will be defined in the SOM. In the case of the determination of UR12788 in urine, the free and conjugated form will be analysed. Full details of the analytical methods used will be described in a separate bioanalytical report.

12.11 Exploratory Sample

An exploratory pharmacogenetic sample will be collected on Day 1 (post-dose). These samples may be analysed in the future to obtain information on genetic factors affecting absorption, distribution, metabolism and excretion of the IMP. Samples will be collected, stored and shipped as detailed in the SOM.

12.12 Volume of Blood Sampling

The total volume of blood that will be drawn from each subject in this study will not exceed 400 mL and is as follows:

Table 7 Volume of Blood to be Drawn from Each Subject

**Assessment**		**Sample Volume (mL)**	**Number of Samples**	**Total Volume (mL)**
PK		7.5	31	232.5
Pharmacogenetic	Exploratory sample	4	1	4
Safety	Biochemistry	5	2	10
	Haematology	4	2	8
	Serology[a]	N/A		
Contingency				145.5
**Total**				400

[a] Serology can be tested from the biochemistry sample; therefore, no extra tube is required.

13. ADVERSE EVENTS

The methods for collecting adverse events are described below.

13.1 Adverse Events

13.1.1 Definitions

The definitions of AEs, adverse drug reactions (ADRs), serious adverse events (SAEs) and SUSARs (Suspected Unexpected Serious Adverse Reactions) are given below.

Adverse event

An AE is the development of an undesirable medical condition or the deterioration of a pre-existing medical condition following or during exposure to a pharmaceutical product, whether or not considered causally related to the product. An undesirable medical condition can be symptoms (e.g., nausea, chest pain), signs (e.g., tachycardia, enlarged liver), or the abnormal results of an investigation (e.g., laboratory findings, electrocardiogram). In clinical studies, an AE can include an undesirable medical condition occurring at any time, from the date informed consent was signed until the end of their participation in a study, i.e., the subject has discontinued or completed the study.

The causality of AEs (i.e., their relationship to study treatment) will be assessed by the Investigator(s) who, in completing the relevant case report form, must answer “yes” or “no” to the question “Do you consider that there is a reasonable possibility that the event may have been caused by any of the following – study medication – other medication?”.

Note that SAEs that could be associated with any study procedure should also be reported.

The following factors should be considered when deciding if there is a “reasonable possibility” that an AE may have been caused by the drug.

- Time Course. Exposure to suspect drug. Has the subject actually received the suspect drug? Did the AE occur in a reasonable temporal relationship to the administration of the suspect drug?

- Consistency with known drug profile. Was the AE consistent with the previous knowledge of the suspect drug (pharmacology and toxicology) or drugs of the same pharmacological class? OR could the AE be anticipated from its pharmacological properties?

- Dechallenge experience. Did the AE resolve or improve on stopping or reducing the dose of the suspect drug?

- No alternative cause. The AE cannot be reasonably explained by another aetiology such as the underlying disease, other drugs, other host or environmental factors.

- Rechallenge experience. Did the AE reoccur if the suspected drug was reintroduced after having been stopped?

- Laboratory tests. A specific laboratory investigation (if performed) has confirmed the relationship?

A “reasonable possibility” could be considered to exist for an AE where one or more of these factors exist.

In contrast, there would not be a “reasonable possibility” of causality if none of the above criteria apply or where there is evidence of exposure and a reasonable time course but any dechallenge (if performed) is negative or ambiguous or there is another more likely cause of the AE.

In difficult cases, other factors could be considered such as:

- Is this a recognised feature of overdose of the drug?

- Is there a known mechanism?

Ambiguous cases should be considered as being a “reasonable possibility” of a causal relationship unless further evidence becomes available to refute this.

Adverse Drug Reaction (ADR)

An ADR is any AE where a causal relationship with the IMP is at least a reasonable possibility.

Serious Adverse Event (SAE)

A serious adverse event is an AE occurring during any study phase (i.e., screening, admission, treatment, washout, or follow-up), and at any dose of the investigational product, comparator or placebo, that fulfils one or more of the following criteria:

- Results in death
- Is immediately life-threatening
- Requires in-patient hospitalisation or prolongation of existing hospitalisation
- Results in persistent or significant disability or incapacity
- Is a congenital abnormality or birth defect
- Is an important medical event that may jeopardise the subject or may require medical intervention to prevent one of the outcomes listed above.

The causality of SAEs (i.e., their relationship to study treatment) will be assessed in the same way as for non-serious AEs.

Note that SAEs that could be associated with any study procedure should also be reported.

Suspected Unexpected Serious Adverse Reactions (SUSAR)

A SUSAR is any SAE where a causal relationship with the IMP is at least a reasonable possibility, but is not listed in the IB.

13.1.2 Recording of adverse events

AEs will be collected from the first administration of investigational product until the end of the study. Serious Adverse Events will be collected during the entire study period.

Any AEs that are unresolved at the subject's last AE assessment in the study (i.e., at discharge) are to be followed up by the Investigator for as long as medically indicated. J. Uriach y Compañía, S.A. retains the right to request additional information for any subject with ongoing AE(s)/SAE(s) at the end of the study, if judged necessary.

AEs spontaneously reported by the subject and/or in response to an open question “Have you had any health problems during the study day/since previous visit?” from the study personnel or revealed by observation will be recorded during the study days at the investigational site.

Findings and values related to physical examinations and measurements of ECG, vital signs, (tympanic temperature, blood pressure, and heart rate) and laboratory parameters will be defined as AEs if they are considered clinically relevant deteriorations compared with baseline and pre-dose values, as judged by the PI.

13.1.3 Assessment of adverse events

The following variables will be recorded for each AE: Symptoms and signs, diagnosis, onset (date and time), resolution (date and time), maximum intensity, frequency, causality (yes or no), outcome, corrective therapy, was the subject withdrawn due to the AE, whether the AE constitutes an SAE or not, and whether the subject was withdrawn due to the AE.

13.1.3.1 Adverse Event Intensity

The intensity rating is defined as:

- mild (awareness of sign or symptom, but easily tolerated)
- moderate (discomfort sufficient to cause interference with normal activities)
- severe (incapacitating, with inability to perform normal activities)

It is important to distinguish between serious and severe AEs. Severity is a measure of intensity whereas seriousness is defined by the criteria in Section 13.1.4. An AE of severe intensity need not necessarily be considered serious. For example, nausea that persists for several hours may be considered severe nausea, but not an SAE. On the other hand, a stroke that results in only a limited degree of disability may be considered a mild stroke but would be an SAE.

In case of an overdose (accidental or deliberate), all symptoms associated with it should be reported as AEs.

13.1.3.2 Adverse Event Causality

For each AE one of the following categories will be selected based on medical judgement, consideration for the definitions below and all contributing factors.

ADR related

Related

A clinical event, including a clinically significantly abnormal laboratory test or other measurement, occurs in a plausible time relationship to drug administration, and which concurrent disease or other drugs or chemicals cannot explain. The response to withdrawal of the drug (*dechallenge) should be clinically plausible. The event must be definitive pharmacologically or phenomenologically, using a satisfactory †rechallenge procedure if necessary.

Probably related

A clinical event, including a clinically significantly abnormal laboratory test or other measurement, with a reasonable time sequence to administration of the drug, unlikely to be attributed to concurrent disease or other drugs or chemicals, and which follows a clinically reasonable response on withdrawal (*dechallenge). †Rechallenge information is not required to fulfil this definition.

Possibly related

A clinical event, including a clinically significantly abnormal laboratory test or other measurement, with a reasonable time sequence to administration of the drug, but which could also be explained by concurrent disease or other drugs or chemicals. Information on drug withdrawal may be lacking or unclear.

Non-ADR related

Unrelated

A clinical event, including a clinically significantly abnormal laboratory test or other measurement, with little or no temporal relationship with drug administration and that may have negative *dechallenge and †rechallenge information. This can typically be explained by

extraneous factors, (e.g., concomitant disease, environmental factors or other drugs or chemicals).

***Dechallenge** is when a drug suspected of causing an AE is discontinued. If the symptoms of the AE disappear partially or completely, within a reasonable time from drug discontinuation, this is termed a positive dechallenge. If the symptoms continue despite withdrawal of the drug, this is termed a negative dechallenge. Note that there are exceptions when an AE does not disappear upon discontinuation of the drug, yet drug-relatedness clearly exists (for example, as in bone marrow suppression, fixed drug eruptions, or tardive dyskinesia).

†**Rechallenge** is when a drug suspected of causing an AE in a specific subject in the past is re-administered to that subject. If the AE recurs upon exposure, this is termed a positive rechallenge. If the AE does not recur, this is termed a negative rechallenge.

13.1.3.3 Outcome

The PI or delegate will follow up all adverse events wherever possible until the symptom has resolved or stabilised.

The date of confirming outcome will be recorded. The course of AEs will be assessed by reference to the following as a guide.

1. Resolved: The AE has resolved and the subject returned to his condition prior to onset.
2. Resolving: The AE has almost resolved and the subject is returning to his condition prior to onset.
3. Not resolved: Even on the final day of observation the AE had not resolved and the subject's condition remained unchanged. In case of death, the subject died of other causes not related to the adverse event from which there was no recovery.
4. Resolved with sequelae: The AE resolved, but the subject has sequelae.
5. Fatal: The subject died. Casual relationship is no object.
6. Unknown: The AE could not be categorised as per above.

13.1.4 Reporting of serious adverse events

If any SAE/SUSAR occurs, the investigators will take appropriate action immediately and will strive to identify the causes of the events.

Any SAE/SUSAR will be notified by the PI to the J. Uriach y Compañía, S.A. Pharmacovigilance Service and the monitor designated by the Sponsor within 24 hours by telephone or fax. Pharmacovigilance (PV) provider's telephone and fax numbers are stated below:

Mr. Alberto Fernández<br>Pharmacovigilance Responsible at J. URIACH y Compañía, S.A.	C/ Camí Reial 51-57,<br><br>08084 Palau de Plegamans<br><br>Tel: 93 – 863 02 88 (Ext: 2288)<br><br>Fax: 93 863 03 13<br><br>Mobile: 629972647

The initial report will be followed up by a full written report within three working days or five calendar days, whichever comes first unless no further information is available. A follow-up report and any subsequent reports will be provided as soon as possible when new information becomes available.

A SUSAR which is fatal or life-threatening must be reported to the Regulatory Authority and the relevant REC by the Sponsor's PV service provider or delegate within 7 days after the Sponsor became aware of the event. If the initial report is incomplete, a complete report must be submitted within 8 days of sending the first response. If significant new information is received by the sponsor on a case already reported, the clock starts again and this should be provided as a follow-up report within 15 days of receipt of the information.

A SUSAR which is not fatal or life-threatening must be reported to the Regulatory Authority and the main REC and in any event within 15 days after the Sponsor first became aware of the event.

SAEs/SUSARs must be recorded and reported whether or not the Investigator considers the SAE/SUSAR to be related to the IMP.

Photocopies of results, consultant report(s), a summary of the outcome of the reaction and the Investigator's opinion of IMP relationship to the SAE/SUSAR will accompany the SAE form if and when available.

If any information relating to the study drug in a study becomes available after the submission of a final protocol to the competent authority which may impact on the conduct of the study, including but not limited to the risk and benefit evaluations underpinning approvals and volunteers consent, J. Uriach y Compañía, S.A. shall notify RPL in writing as soon as practically possible and the parties will agree, in writing, what steps need to be taken, if any.

14. QUALITY ASSURANCE AND QUALITY CONTROL

To ensure GCP compliance and compliance with all applicable regulatory requirements, the Sponsor or RPL may conduct a quality assurance audit. A regulatory inspection of this study may be carried out by regulatory agencies. Such audits/inspections can occur at any time during or after completion of the study. If an audit or inspection occurs, the PI and institution agree to allow the auditor/inspector direct access to all relevant documents and to allocate their time and the time of their staff to the auditor/inspector to discuss findings and any relevant issues.

Quality control (QC) procedures at RPL will be implemented to ensure data recorded into the CRFs are accurate before CRFs are sent for data entry purposes, QC checks will be carried out on critical phases in the execution of the study. These control checks will be carried out according to the relevant standard operating procedures. Records of these procedures will be documented and available for review.

14.1 Monitoring

The monitoring of this study will be performed by the Sponsor's Monitor(s) or a designee in accordance with the principles of GCP as laid out in the International Conference on Harmonisation (ICH) document "Good Clinical Practice: Consolidated Guideline".

The person designated by J. Uriach y Compañía, S.A. will have regular contact with the study site, including visits to:

- Provide information and support to the Investigators
- Confirm that facilities remain acceptable
- Confirm that the investigational team is adhering to the protocol, that data are being accurately and timely recorded in the CRFs, that biological samples are handled in accordance with the SOM and that investigational product accountability checks are being performed.
- Perform source data verification (a comparison of the data in the CRFs with the subject's medical records at the hospital or practice, and other records relevant to the study) including verification of informed consent of participating subjects. This will require direct access to all original records for each subject (e.g., clinic charts).
- Ensure withdrawal of informed consent to the use of the subject's biological samples is reported and biological samples are identified and disposed of/destroyed accordingly, and the action is documented, and reported to the subject.

15. STATISTICAL EVALUATION

15.1 Statistical Analysis Plan

A Statistical Analysis Plan (SAP) will be written after finalising the protocol and before database lock. The specifications in this document will detail the implementation of all the planned statistical analyses in accordance with the principal features stated in the protocol.

15.2 Analysis Sets

15.2.1 Safety set

All subjects who receive at least a single dose of the study medication.

15.2.2 PK set

All subjects who receive at least single dose of the study medication and who have evaluable PK data.

15.2.3 PD set

All subjects who receive at least single dose of the study medication and who have evaluable PD data.

15.3 PK Parameters

PK parameters will be derived by RPL from the relevant plasma concentration data of Rupatadine and its two main metabolites UR 12790 and UR 12788, by non-compartmental analysis using SAS™ v9.2 or above.

Single dose pharmacokinetics: The following PK parameters will be derived to assess the single dose PK and the overall exposure to Rupatadine and the two main metabolites, UR 12790 and UR 12788:

Plasma:

- Maximum plasma concentration (C_{max})
- Time to reach maximum plasma concentration (T_{max})
- Area under the plasma concentration vs time curve from zero to 24 hours post-last dose (AUC_{τ}, where τ = 24h post dose on Day 1)
- Area under the plasma concentration vs time curve from zero to infinity ($AUC_{0-\infty}$)
- Half-life ($t_{½}$)
- Apparent volume of distribution (Vz/F) - for Rupatadine only
- Oral plasma clearance (CL/F) - for Rupatadine only

Urine:

- Amount of drug excreted in the urine (Ae)
- Renal clearance (CLR)

Trough plasma concentrations: Pre-dose plasma samples will be taken on Days: 2 (the same sample as 24 hours post dosing on Day 1), 3 and 4 in order to determine the minimum plasma concentration (C_{min}) for Rupatadine and its two main metabolites, UR 12790 and UR 12788.

Steady state pharmacokinetics: The following PK parameters will be derived in order to assess the steady state PK and the overall exposure to Rupatadine and the two main metabolites:

Plasma:

- C_{max}
- T_{max}
- AUC_{τ}, where τ = 24h post dose on Day 5
- Area under the plasma concentration vs time curve from time zero to the last quantifiable concentration (AUC_{0-t})
- $AUC_{0-\infty}$
- $t_{½}$
- Minimum plasma concentration (C_{min})

- Average plasma concentration (C_{avg})
- Mean residence time (MRT)
- Vz/F - for Rupatadine only
- CL/F - for Rupatadine only
- Accumulation ratio (Rac)

Urine:

- Ae
- CLR

PK parameter definitions:

C_{max}	The observed maximum drug plasma concentration
T_{max}	Time to reach maximum plasma concentration C_{max}
AUC_{0-t}	Area under the plasma concentration vs time curve from time zero to the last quantifiable concentration (C_{last}), calculated by the linear up-log down trapezoidal method.
$AUC_{0-\infty}$	Area under the plasma concentration vs time curve from zero to infinity. The total area under the plasma concentration vs time curve, calculated by AUC_{0-t} + $AUC_{t-\infty}$, where $AUC_{t-\infty}$ is the residual area under the plasma concentration vs. time curve, extrapolated by C_{last}/λ_z (λ_z is the elimination rate constant estimated from individual linear regression of the terminal part of the log concentration vs time curve).
AUC_{τ},	Area under the plasma concentration vs time curve from time of last dose to 24 hours post-last dose, calculated by the linear up-log down trapezoidal method.
λ_z	The elimination rate constant estimated from individual linear regression of the terminal part of the log concentration vs time curve
$t_{½}$	The terminal elimination half-life, calculated by $0.693/\lambda_z$ (λ_z is the elimination rate constant estimated from individual linear regression of the terminal part of the log concentration vs time curve)
C_{min}	The observed minimum drug plasma concentration
C_{avg}	The observed average drug plasma concentration
MRT	Mean residence time, calculated as follows: MRT = $AUMC_{t-\infty}$/ $AUC_{t-\infty}$, where $AUMC_{t-\infty}$ is the area under the moment curve and t = 0.
Vz/F	Apparent volume of distribution, calculated as follows: Vz/F = [Dose/ AUC(0-inf)]/ λz
Rac	The accumulation ratio will be calculated as follows: Rac = AUC_{τ}, Day 5 / AUC_{τ}, Day 1
CL/F	Oral plasma clearance, calculated as follows: CL/F = Dose/ $AUC_{0-\infty}$
Ae	Amount of drug excreted in the urine (Ae), calculated as follows: Ae = urine concentration x urine volume (cumulative)
CLR	The renal clearance will be calculated as follows: CLR = Ae Day 1 / AUC_{τ}, Day 1; CLR = Ae Day5 / AUC_{τ}, Day 5.

Actual sampling times will be used for all calculations of the PK parameters. If there is any doubt in the actual time a sample was taken, then the scheduled time will be used. Special consideration will be given to the estimation of λ_z and corresponding $t_{½}$ values. Values of λ_z will be calculated from a minimum of three data points, wherever possible. Any values below the LLOQ of the assay before the T_{max} will be assumed to be zero. Values below the LLOQ which occur after the T_{max} will be ignored.

15.4 PD Parameters

PD evaluation will include the assessments of the following cognitive parameters:

- Rapid Visual Information Processing (RVP)
- Reaction Time (RTI)
- Spatial Working Memory (SWM)
- Visual Analogue Scales (VAS)

15.5 Safety Parameters

Safety assessments will include standard laboratory safety tests (haematology, biochemistry and urinalysis), vital signs (blood pressure and heart rate), physical examinations, 12-lead ECG and AE monitoring.

15.6 Statistical analysis

15.6.1 PK Analysis

Pharmacokinetic data will be listed for each subject, along with summary statistics including arithmetic and geometric means, standard deviations, minimum, maximum and median values, and coefficients of variation.

Dose proportionality will be analysed with a mixed effect model using the logarithm of a PK parameter as responsible variable and the logarithm of the dose as fixed factor and subject as random effect. Based on the mixed effect model, the dose proportionality coefficient and its 2-sided 95% confidence interval will be estimated. Dose proportionality will be declared if 95% confidence interval contains 1. The above model will be applied to $AUC_{0-\infty}$, AUC_{τ} and C_{max}, after a single dose and at stead state.

15.6.2 PD Analysis

Results of cognitive tests will be listed for each subject, along with summary statistics including arithmetic and geometric means, standard deviations, minimum, maximum and median values, and coefficients of variation. Further analysis may be performed in an exploratory manner and will be documented in the SAP, as appropriate.

15.6.3 Safety Data Analysis

15.6.3.1 Adverse events

AEs will be coded and reported according to MedDRA, version 15.1 or higher including system organ class and preferred term. Treatment emergent AEs will be assigned to the dose received preceding event onset. All AEs will be included in the data listings. Only treatment emergent AEs (i.e., commencing after dosing with the IMP) will be included in the summary tables.

The number and percentage of subjects reporting AEs, SAEs, IMP-related AEs and AEs leading to withdrawal will be summarised.

15.6.3.2 Laboratory parameters

Laboratory test results will be listed and compared to laboratory reference ranges, with those values outside of the applicable range flagged as high (H) or low (L). The quantitative laboratory data, along with changes from baseline will be summarised using descriptive statistics. The qualitative urinalysis data will be summarised at each time point by dose.

15.6.3.3 Vital signs

Vital signs data (systolic and diastolic blood pressure, heart rate and tympanic body temperature) will be listed for individual subjects. Summary statistics (n, arithmetic mean, median, minimum, and maximum) will be calculated for each parameter by treatment and time.

15.6.3.4 Standard 12-lead ECG parameters

<u>Automated (machine generated) ECG values</u>

Automated (ECG machine generated) values for ECG parameters (RR, PR, QRS, QT, QTcB, QTcF intervals and HR) will be listed for individual subjects. Out-of- range ECG values will be flagged as high (H) or low (L). Summary statistics (n, arithmetic mean, median, minimum, and maximum) will be calculated for each parameter by treatment and time.

<u>Cardiologist over-read ECG values</u>

Heart Rate Correction: Fridericia's QT correction formula will be used to estimate the QTc interval.

Concentration Effect Relationship: Plots of the differences with 90% CIs between plasma Rupatadine, desloratadine and hydroxylatedesloratadine concentrations versus baseline (Day -1 data) and placebo over time will be produced for all analyses.

Assay sensitivity for the ECG analyses will be assessed by calculating the food effect in a time course effect analysis.

Further details on the ECG analyses will be provided in the SAP as appropriate.

15.7 Handling of Missing and Incomplete Data

Unrecorded values will be treated as missing. The appropriateness of the method(s) described for handling missing data will be reassessed and documented at the blind data review prior to database lock. Depending on the extent of missing values, further

investigation may be made into the sensitivity of the analysis results to the method(s) specified.

15.8 Sample Size Considerations

Due to the exploratory nature of the study, the sample size is not based on formal statistical considerations. It is based on experience from previous similar Phase I studies with other compounds and deemed adequate to achieve study objectives.

16. DATA MANAGEMENT

Data Management will be performed by the Data Management department of RPL. The data management process will be described in detail in the Data Handling Protocol (DHP).

The RPL Data Management department will be responsible for developing and maintaining the DHP; setting-up and validating the clinical study database; programming validation checks; entering data into the study database; reviewing data for accuracy, completeness and consistency between the CRF and the database; and verifying adherence to the clinical pharmacology study protocol and the DHP.

The study database will be constructed using Oracle Clinical version 4.5 based on the CRF data, using double independent data entry with second entry verification.

Safety laboratory data will be uploaded into the database as an electronic data transfer according to the validated transfer specification.

Data queries will be generated and resolved according to the DHP. All queries are documented individually on a Data Clarification Form (DCF) which is generated in Oracle Clinical. The DCF is a form designed to maintain an audit trail of modifications of the data in the clinical database and the justification for those modifications. All queries are resolved with the assistance of RPL clinical staff.

Any SAEs in the clinical database will be reconciled with the safety database.

The database is locked after all clinical data queries are resolved, the database is declared clean and the final error rate and QC checks are confirmed as acceptable.

Standard SAS™ datasets are generated from the locked study database ready for analyses. A complete audit trail of all corrections is available for inspection. The datasets will be transferred to Statistician according to the Data Transfer Specification.

The database will be frozen after TFLs are declared final and final Clinical Study Report is signed off.

16.1 Case Report Forms

Case Report Forms (CRFs) will be used to record the data in the study. Data should be recorded legibly onto the CRFs in black ballpoint pen. Correction fluid or covering labels must not be used.

The designated J. Uriach y Compañía, S.A. monitor will check data at the monitoring visits to the study site. The Investigator will ensure that the data in the CRFs are accurate, complete, and legible.

Data from the completed CRFs will be entered into RPL's clinical study database and validated under the direction of the Data Manager. Data from screening failures (subjects who signed consent to take part in the study but were not randomised) will not be entered into the clinical study database. Any missing, impossible (inconsistent with human life), or inconsistent recordings in the CRFs will be referred back to the Investigator using a DCF and be documented for each individual subject before clean file status is declared.

17. SPONSOR'S AND INVESTIGATOR'S RESPONSIBILITIES

17.1 Sponsor's Responsibilities

17.1.1 GCP compliance

J. Uriach y Compañía, S.A. and any third party to whom aspects of the study management or monitoring have been delegated will undertake their roles for this study in compliance with all applicable regulations and ICH GCP Guidelines.

Visits to Investigator sites will be conducted by representatives of J. Uriach y Compañía, S.A. to inspect study data, subjects' medical records, and CRFs in accordance with current GCP and the respective local and national government regulations and guidelines. Records and data may additionally be reviewed by auditors or by regulatory authorities.

17.1.2 Regulatory approval

RPL on behalf of J. Uriach y Compañía, S.A. will ensure that Local Regulatory Authority requirements are met before the start of the study.

17.1.3 Protocol management

All protocols and amendments will be prepared by J. Uriach y Compañía, S.A. and/or RPL. If it becomes necessary to issue a protocol amendment during the course of the study, J. Uriach y Compañía, S.A. will notify the Investigator and collect documented Investigator Agreement to the amendment.

17.1.4 End of trial notification

RPL on behalf of J. Uriach y Compañía, S.A. will submit an end of trial notification to the competent authority of the Member State within 90 days of the end of the trial in accordance with EU Directive 2001/20/EC. The PI will be responsible for submitting these to the REC within 90 days of the end of the trial.

For the purposes of this notification, the end of the trial will be defined as the last subject/last visit.

17.1.5 Submission of summary of clinical trial report to competent authorities of member states concerned and RECs

RPL on behalf of J. Uriach y Compañía, S.A. will provide a summary of the clinical trial report within one year of the end of the complete trial to the competent authority of the Member

State concerned as required by the regulatory requirement and to comply with the Community guideline on Good Clinical Practice.

17.2 Investigator's Responsibilities

17.2.1 GCP compliance

The Investigator must undertake to perform the study in accordance with ICH GCP Guidelines, EU Directive 2001/20/EC, and the applicable regulatory requirements.

It is the Investigator's responsibility to ensure that adequate time and appropriate resources are available at the study site prior to commitment to participate in this study. The Investigator should also be able to estimate or demonstrate a potential for recruiting the required number of suitable subjects within the agreed recruitment period.

The Investigator will maintain a list of appropriately qualified persons to whom the Investigator has delegated significant trial-related tasks. An up-to-date copy of the *curriculum vitae* for the Investigator, Sub-investigator(s), and essential study staff will be provided to J. Uriach y Compañía, S.A. (or designee) before starting the study.

Agreement with the final Clinical Study Report will be documented by the dated signature of the PI, in compliance with Directive 75/318/EC, Directive 2001/83/EC, and ICH E3.

17.2.2 Regulatory approval

RPL on behalf of J. Uriach y Compañía, S.A. will be responsible for the preparation, submission, and confirmation of receipt of any Regulatory Authority approvals. J. Uriach y Compañía, S.A. will be responsible for giving a green light for release of investigational product for shipment to the study site.

17.2.3 Indemnity/liability and insurance

J. Uriach y Compañía, S.A. will adhere to the recommendations of the Association of British Pharmaceutical Industry (ABPI) Guidelines. A copy of the Indemnity document will be supplied to the Investigator before study initiation.

J. Uriach y Compañía, S.A. will ensure that suitable insurance cover is in place prior to the start of the study. For this purpose they will provide RPL with a statement of insurance cover document from and an insurance certificate will be supplied to RPL.

17.2.4 Protocol adherence and investigator agreement

The PI must adhere to the protocol as detailed in this document. The Investigator will be responsible for enrolling only those subjects who have met protocol eligibility criteria. The Investigators will be required to sign an Investigator Agreement to confirm acceptance and willingness to comply with the study protocol.

17.2.5 Pharmacovigilance

J. Uriach y Compañía, S.A. is responsible for fulfilling all obligations (expedited safety reporting, e.g. SUSARs; periodic reporting, e.g. annual reports) regarding notification of Competent Authorities and Ethics Committees according to the current international legislation. The information for investigators is also the duty of J. Uriach y Compañía, S.A.

17.2.6 Documentation and retention of records

After completion of the study, all documents and data relating to the study will be kept in an orderly manner by the Investigator in a secure file and/or electronically. This file will be available for inspection by the Sponsor or their representatives. Essential documents must be retained for two years after the final marketing approval in an ICH region or at least two years have elapsed since the discontinuation of clinical development of the IMP. The Investigator must contact the Sponsor before destroying any study-related documentation and it is the responsibility of the Sponsor to inform the investigative site of when these documents can be destroyed. In addition, all subject records and other source documentation will be kept for a longer period if required by the applicable regulatory requirements.

17.3 Ethical considerations

This protocol complies with the principles of the World Medical Assembly (Helsinki 1964) and subsequent amendments.

17.3.1 Informed consent

The informed consent is a process by which a subject voluntarily confirms his/her willingness to participate in a clinical trial. It is the responsibility of the Investigator to obtain a written informed consent from each subject participating in the trial, after explanation of the aims, methods, benefits and potential hazards of the trial and in accordance with Good Clinical Practice requirement (CPMP/ICH/135/95) and Directive 2001/20/EC. The information sheet and consent form given to the subject should be kept clear, relevant and understandable to a lay person and it should be written in the language the subject understands well. The measures taken to safeguard his/her privacy and the protection of personal data should be described. The formalities for collection, storage and management of these personal data must be fully described on the informed consent in form of request for their specific utilization to be authorized by the subjects participating in the study.

The Investigator should provide the subject with sufficient time to decide whether or not participate in the trial. The subject should be also provided with a contact point where he/she may obtain further information.

The subject information sheet, consent form and form for the protection of personal data should be translated in local language and adapted to local requirement (e.g., in respect to data protection), as necessary, before submission to the relevant Ethics Committee for approval. Only the approved version can be used in the centre. Each version should be identified with the date.

The signatures of the consent form and the protection of personal data form must be obtained before any trial specific procedures are performed on the subject. The Investigator must keep one form personally signed and dated by the subject on file and a copy of it must be delivered to the subject. The informed consent must be documented in the case report form. The subject should be informed if new information becomes available that might be relevant to the subject’s willingness to continue participation in the trial.

17.3.2 Research Ethics Committee (REC) approval

It is the responsibility of the Investigator to submit this protocol, the informed consent document (approved by J. Uriach y Compañía, S.A.), and all relevant supporting information, to the REC for review. In addition, study specific advertisements must be approved by the REC prior to use at the site.

Prior to implementing substantial changes in the study, J. Uriach y Compañía, S.A. and the REC must also approve any revised informed consent documents and substantial amendments to the protocol.

On the approval letter, the trial (title, protocol number, and version), the documents reviewed (protocol, informed consent material) and the date of review and actions taken should be clearly stated.

17.4 Confidentiality

Data collected during this study may be used to support the development, registration, or marketing of medicinal product. J. Uriach y Compañía, S.A. will control all data collected during the study, and will abide by the EU Directive on Data Privacy concerning the processing and use of subjects' personal data. For the purpose of data privacy legislation, J. Uriach y Compañía, S.A. will be the data controller.

After subjects have consented to take part in the study, their medical records and the data collected during the study will be reviewed by J. Uriach y Compañía, S.A. and/or its representatives. These records and data may, in addition, be reviewed by the following: independent auditors who validate the data on behalf of J. Uriach y Compañía, S.A.; national or local regulatory authorities, and the REC which gave its approval for this study to proceed.

Although subjects will be known by a unique number, their initials and date of birth will also be collected and used to assist J. Uriach y Compañía, S.A. to verify the accuracy of the data, for example, that the results of study assessments are assigned to the correct subject. The results of this study containing the unique number, initials, date of birth, and relevant medical information including ethnicity may be recorded and transferred to and used in other countries throughout the world, which may not afford the same level of protection that applies within the EU. The purpose of any such transfer would be to support regulatory submissions made by J. Uriach y Compañía, S.A. in such countries.

17.5 Publication Policy

J. Uriach y Compañía, S.A. has no objection to publication by Richmond Pharmacology of any information collected or generated by Richmond Pharmacology subject to the safeguards set out below. To ensure there is no inadvertent disclosure of Confidential Information, unprotected inventions, or Study Results, Richmond Pharmacology will provide J. Uriach y Compañía, S.A. with an opportunity to review any proposed publication or other type of disclosure before it is submitted or otherwise disclosed. Any such proposed publication will require the written consent of J. Uriach y Compañía, S.A. Proposed Publications shall not include confidential information other than the study results or any personal data on any subject, such as name or initials.

Richmond Pharmacology will provide manuscripts or abstracts to J. Uriach y Compañía, S.A. at least sixty (60) days before they are submitted for publication or presentation. If any patent filing is required to protect J. Uriach y Compañía, S.A. intellectual property rights, Richmond Pharmacology shall delay submission of the manuscript or abstract for a period not to exceed an additional 4 months.

Richmond Pharmacology will provide the full text of any other intended disclosure (poster presentation, invited speaker or guest lecturer presentation, etc) to J. Uriach y Compañía, S.A. at least ninety (90) days before the date of proposed disclosure.

Richmond Pharmacology will, on request, remove from any intended disclosure any previously undisclosed J. Uriach y Compañía, S.A. Confidential Information (other than the Study results themselves) before disclosure.

18. REFERENCES

1. Summary of Product Characteristics (SmPC) for Rupafin 10mg Tablets. Available at: http://www.medicines.org.uk/emc/medicine/21780/SPC/Rupafin+10mg+Tablets/
2. Rupatadina (INN) Fumarate Clinical Investigator Brochure. Version 8, July 2011. Grupo Uriach.
